# Single-stranded nucleic acid binding enhances the in vitro catalytic activity of Chikungunya virus nsP2 protease

**DOI:** 10.1016/j.bbadva.2026.100198

**Published:** 2026-07-09

**Authors:** Mohammadamin Mastalipour, Danilo Silva Olivier, Mônika Apareçida Coronado, Andrew J. Dingley, Ruth Anasthasia Siahaan, Alissa Drees, Christian Ahlers, Markus Fischer, Dieter Willbold, Raphael Josef Eberle

**Affiliations:** a Institut für Physikalische Biologie, Heinrich-Heine-Universität Düsseldorf, Düsseldorf, Germany; b Integrated Sciences Center, Campus Cimba, Federal University of Tocantins, Araguaína, 77824-838, TO, Brazil; c Institut für Biologische Informationsprozesse (IBI-7), Forschungszentrum Jülich, Jülich, Germany; d Faculty of Chemistry and Biotechnology, FH Aachen, Campus Jülich, Germany; e Hamburg School of Food Science, Institute of Food Chemistry, University of Hamburg, Hamburg, Germany; f Institut für Biochemische Pflanzenphysiologie, Heinrich-Heine-Universität Düsseldorf, Düsseldorf, Germany

**Keywords:** Chikungunya virus, nsP2 protease (nsP2^pro^), Single-stranded nucleic acid, Protein–nucleic acid interaction, Aptamers, Viral replication

## Abstract

Chikungunya virus (CHIKV) is an emerging arbovirus whose replication relies on the multifunctional nonstructural protein 2 (nsP2), particularly its viral protease (nsP2^pro^), which is essential for polyprotein processing. In this study, we investigated how interactions with nucleic acids influence nsP2^pro^ activity. Using High-Throughput Sequencing–Fluorescent Ligand Interaction Profiling (HiTS-FLIP), we identified specific single-stranded DNA aptamers that enhanced nsP2^pro^ activity. Additionally, both random single-stranded DNA and single-stranded RNA were found to stimulate protease activity, whereas double-stranded DNA showed no such effect. Circular dichroism spectroscopy and secondary structure predictions confirmed that the identified aptamers adopt stable folded conformations. Similarly, structured RNA sequences were also capable of promoting protease activity. The observed stimulatory effect depended on the nucleic acid strand type, length, and buffer conditions, suggesting the involvement of electrostatic interactions. NMR experiments demonstrated that nsP2^pro^ interacts with single-stranded DNA, accompanied by changes in its conformational dynamics upon binding. Molecular dynamic simulations of nsP2^pro^-RNA and -DNA complexes revealed that these nucleic acids interact specifically with the nsP2^pro^ methyltransferase domain and induce an opening process in the protease active site. Our findings provide novel insights into the regulation of nsP2^pro^, enhancing our understanding of CHIKV replication mechanisms, and may guide future antiviral development strategies.

## Highlights

• Nucleic acids are identified as previously unrecognized modulators of in vitro CHIKV nsP2 protease activity.
• DNA aptamers, DNA and RNA molecules promote nsP2 proteolytic activity through sequence- and length-dependent effects.
• Biophysical and in silico analyses demonstrate that nucleic acid binding alters nsP2 conformational dynamics and active-site accessibility.
• These findings reveal a new layer of regulation in alphavirus replication and suggest alternative strategies for antiviral intervention.

## 1 Introduction

Changes in climate and environmental conditions, including rising temperatures, create favorable environments for the proliferation and spread of vectors such as mosquitoes [1]. This, in turn, facilitates the transmission of arthropod-borne viruses to areas where they were previously not endemic, including Chikungunya virus (CHIKV), Dengue virus (DENV), and other tropical infections [2]. Chikungunya, a member of the *Alphavirus* genus within the *Togaviridae* family [3,4], has caused outbreaks across Asia, and South and Central Africa, infected over 3 million people worldwide [5]. Currently, health officials in China are tackling an outbreak of Chikungunya virus in Guangdong province, which has resulted in >7000 reported cases since June 2025 [6]. Approximately 39% of the world’s population lives in endemic regions and remains at risk of infection [7]. CHIKV can cause a variety of clinical symptoms, including high fever, joint pain, skin rash, and arthralgia, as well as complications such as cardiac and neurological issues [8,9]. The infection can progress to a chronic phase characterized by persistent pain and joint inflammation [10]. Patients in this phase may also experience depression, memory loss, and sleep disturbances [10,11], which contribute to disability and significantly reduce quality of life [12]. CHIKV is a positive-sense, single-stranded RNA virus whose genome encodes five structural proteins, including envelope (E) and capsid proteins, along with four non-structural proteins (nsP1-nsP4) [13,14]. Among these, nsP2 is a multifunctional protein essential for viral infection [15]. It contains an N-terminal RNA helicase with both nucleotide triphosphatase (NTPase) and RNA triphosphatase activities, and a C-terminal papain-like cysteine protease domain (nsp2^pro^) responsible for polyprotein processing, followed by a Ftsj methyltransferase (MTase)-like domain [15,16]. Law et al. 2019 provided a structural analysis of the CHIKV nsP2 helicase domain bound to RNA [17]. Besides its proteolytic activity, nsP2^pro^ interacts with host proteins, further facilitating viral replication. Notably, studies have demonstrated that nsP2^pro^ migrates to the cell nucleus, although the precise biological significance of this localization remains unclear [18]. The interferon response is a critical first-line defense mechanism of the innate immune system against viral infections, but it can be suppressed by viral factors [19]. Specifically, the methyltransferase domain of CHIKV nsP2^pro^ is known to downregulate the interferon response by inhibiting the JAK/STAT signaling pathway, which is essential for interferon activation [20]. Furthermore, this domain disrupts MHC-I antigen presentation, enabling the virus to evade detection by CD8^+^ T cells [21]. Given that many virus proteases are activated upon interaction with cofactor proteins, these proteases interact with virus and host nucleic acids (DNA or RNA) in several ways, primarily to process viral polyproteins and regulate host cell functions. These interactions are crucial for viral replication, pathogenesis, and immune evasion. Table 1 summarizes reported virus protease-nucleic acid interactions.

**Table 1:** Summary of virus proteases interacting with nucleic acids.

Virus	Virus protease	Nucleic acid	Binding/Activation/Inhibition	Refs.
Coxsackievirus	3C^pro^	Virus RNA	Binding	[22]
Foamy virus	Foamy virus protease	Virus RNA	Activation	[23]
Hepatitis A virus	3C^pro^	Synthetic DNA	Inhibition	[24]
Hepatitis C virus	NS3 protease	Virus RNA	Binding	[25]
Hepatitis C virus	NS3 protease	RNA aptamer	Inhibition	[26]
Human immunodeficiency virus type 1	HIV-1 PR	Virus RNA	Activation	[27]
Human immunodeficiency virus type 1	HIV-1 PR	RNA aptamer	Inhibition	[28]
Poliovirus	3C^pro^	Virus RNA	Activation	[29]
Seneca Valley virus	3C^pro^	Host DNA	Activation	[30]

Aptamer-guided mapping of nucleic acid interaction surfaces on viral proteases represents a powerful strategy to reveal information about functional binding hotspots and dynamic regulatory regions that influence catalytic activity, providing a framework for dissecting viral replication mechanisms and identifying novel therapeutic targets (Tuerk & Gold, 1990; Stoltenburg et al., 2007; Keefe et al., 2010). Since its introduction in 1990 [3,4], aptamer selection has primarily relied on Systematic Evolution of Ligands by Exponential Enrichment (SELEX). However, limited control over the selection process results in modest success rates (20–30%) and prolonged experimental timelines [5,6]. High-Throughput Sequencing–Fluorescent Ligand Interaction Profiling (HiTS-FLIP) is a approach that combines next-generation sequencing flow cells to quantitatively measure interactions between millions of immobilized nucleic acid sequences and fluorescently labeled ligands. By coupling sequence information with fluorescence-based binding measurements, HiTS-FLIP enables comprehensive mapping of sequence–function relationships and high-resolution characterization of molecular recognition landscapes.. This strategy significantly improves the efficiency of aptamer identification [[7], [8], [9]].

In this study, we explore how nucleic acids influence the catalytic activity of nsP2^pro^ to better understand the regulation of its enzymatic function. Through HiTS-FLIP, we identified specific DNA aptamers that bind to nsP2^pro^ and evaluated their effects on its proteolytic activity. Additionally, we demonstrate that random single-stranded RNA (ssRNA) and single-stranded DNA (ssDNA) also modulate nsP2^pro^ function, and that nsP2^pro^ directly interacts with single-stranded nucleic acids. Finally, *in silico* analyses and MD simulations were conducted to predict the potential binding sites of these nucleic acids on nsP2^pro^.

## 2 Materials and methods

### 2.1 Expression and purification of nsP2^pro^

All experimental analyses were performed with a recombinant nsP2 construct containing the protease and methyltransferase domains (nsP2^pro^: residues 470–798), which together constitute the catalytically relevant region examined in this study. The protein was expressed and purified as previously described [31,32]. For NMR experiments, ^15^N-labeled nsP2^pro^ was expressed in M9 minimal medium using a reported protocol [33].

### 2.2 High-throughput sequencing–fluorescent ligand interaction profiling (HiTS-FLIP)

The aptamer selection was performed by HiTS-FLIP as described previously [34]. In specific, we sequenced a random library (5′-TCGCACATTCCGCTTCTACC—N_50—_CGTAAGTCCGTGTGTGCGAA-3′, acquired PAGE-purified and hand-mixed from Integrated DNA Technologies Inc., Coralville, IA, USA) using a v2 Nano 300 cycle MiSeq kit (Illumina Inc., San Diego, CA, USA) after spiking in 12% phiX (Illumina Inc., San Diego, CA, USA) and 1.25% fiducial mark oligo (acquired from Integrated DNA Technologies Inc., Coralville, IA, USA). Of the 1682,200 clusters, 84.5% passed filters. For the aptamer selection via HiTS-FLIP, CHIKV nsP2^pro^ was labelled using AF647-NHS-ester (Lumiprobe GmbH, Hannover, Germany), which primarily binds to lysine side chains and the α-amino group at the N-terminus of the protein, with a labelling efficiency of approximately 1 dye molecule per protein. Subsequent to sequencing, the labeled protein was introduced to the flow cell of a modified MiSeq sequencer (Illumina Inc.) at concentrations of 29.8 pM, 149 pM, 745 pM, 3.73 nM, 18.7 nM, 93.2 nM, and 466 nM in 1x PBS + 0.1% Tween20 (pH 7.4, Sigma-Aldrich Corp., St. Louis, MO, USA) via an external valve (C25Z-31812EUHB, Valco Instruments Co. Inc., Houston, TX, USA; VICI). On the flow cell, each protein concentration was incubated for 30 min at 37 °C. For each DNA cluster, fluorescence signals were measured and normalized to reflect the amount of AF647-NHS-ester labelled CHIKV nsP2^pro^ bound at each concentration. Binding curves for each cluster were generated by plotting fluorescence intensity against protein concentration, and K_D_ values were determined using a one-site binding model with Hill-fit analysis in MATLAB R2022b (The MathWorks Inc., Natick, MA, USA). The resulting apparent K_D_ values represent the equilibrium binding affinities under the specific conditions of the flow cell assay [34]. Out of the 910,150 distinct aptamer library sequences displayed on the flow cell, after filtering the data as described previously [34] the ten aptamer candidates with the highest affinity were chosen for further analysis.

### 2.3 Nucleic acids

All DNA aptamers and RNA oligonucleotides were purchased in lyophilized form from Integrated DNA Technologies, Inc. (Coralville, Iowa, USA). DNA aptamers were purified by standard desalting, while RNA oligonucleotides were purified by RNase-free high-performance liquid chromatography (HPLC). Two RNA oligonucleotides a 5-mer and a 10-mer sequences, randomly derived from the CHIKV genome and were used in the study (S1 Table). Additionally, a single-stranded DNA (ssDNA) oligonucleotide (5′-CGTCGCTATA-3′) with the same sequence as RAC2 was purchased from Integrated DNA Technologies (Coralville, Iowa, USA) and included in the experiments. As controls regarding the specificity, double-stranded DNA (dsDNA) with a random sequence was isolated from brain tissue of TgM83^+/−^ mice [35] using the DNeasy Blood & Tissue Kit (Qiagen GmbH, Hilden Germany), which is optimized for genomic DNA extraction. The double-stranded DNA was provided by Sara Reithoffer at the Institut für Physikalische Biologie, Heinrich-Heine-Universität Düsseldorf. A random single-stranded DNA (5′-TGACCATGGAGCCTGCCGTCTACTTCAAG-3′) was obtained from Sigma-Aldrich (St. Louis, MO, USA) and kindly provided by Dr. Jeannine Mohrlüder, from the Institut für Biologische Informationsprozesse, Strukturbiochemie (IBI-7), Forschungszentrum Jülich.

### 2.4 Enzymatic assay

To assess the activity of CHIKV nsP2^pro^ and the effects of nucleic acids, a FRET-based assay was performed using a synthesized fluorogenic peptide substrate, DABCYL-Arg-Ala-Gly-Gly-↓Tyr-Ile-Phe-Ser-EDANS (BACHEM, Bubendorf, Switzerland) [31]. The enzymatic assay was conducted in a 96-well plate with a final reaction volume of 100 µL per well. Unless otherwise stated, 1 × PBS (pH 7.5) was used as the standard assay buffer condition.

Each well contained 10 µM nsP2^pro^, 9 µM fluorogenic substrate, and nucleic acids at a protease-to-nucleic acid molar ratio of either 1:1 (10 µM) or 1:3 (30 µM). Fluorescence intensities were measured (excitation at 340 nm, emission at 490 nm) every 30 s for 30 min at 37 °C. Protease activity assays were conducted under the indicated conditions, and reaction progress was monitored over time to verify linearity. For comparative analyses, activity values obtained at 20 min were used, as the reaction remained within the linear range throughout this period (Fig. S1).

To assess the influence of buffer conditions on nsP2^pro^ activity, a separate experiment was conducted using three different buffer environments: 20 mM Phosphate buffer (pH 7.5), 20 mM Bis-Tris propane (pH 7.5), and 400 mM NaCl in Bis-Tris propane buffer (pH 7.5).

The activity and effects of nucleic acids on the protease were calculated using the following Eq. (1):

$$\text{Protease}\;\text{activity}\;\%=\;\frac{\text{Fluorescence}\;\text{intensity}\;\text{with}\;\text{nucleic}\;\text{acid}}{\text{Fluorescence}\;\text{intensity}\;\text{without}\;\text{nucleic}\;\text{acid}}\;\times \;100$$

All assays were carried out in technical triplicates, and the resulting data were analyzed and presented as mean ± standard deviation (SD).

### 2.5 Structure predication of DNA aptamers

The secondary structures of DNA aptamers were predicted using the DINAMelt Server – Quikfold (https://www.unafold.org/quikfold.php) [36]. The Fast Folding – Energies & Structures algorithm was used to determine the most thermodynamically stable conformations. Predictions were carried out at 18 °C with two different Na⁺ concentrations: 137 mM and 10 mM. The sequence type was set to linear, and all other parameters were kept at their default values as provided by the server, including 5% suboptimality, a default window size, a maximum of 50 suboptimal foldings, and no limit on the distance between base-paired nucleotides.

### 2.6 CD spectroscopy

DNA and RNA samples were separately prepared in ddH₂O and 1 × PBS, respectively, to final concentrations of 30 µM for DNA aptamers and 50 µM for RNAs, enabling subsequent secondary structure analysis Circular Dichroism (CD) spectra were recorded at 18 °C using a 0.1 mm pathlength cuvette (Hellma GmbH & Co.KG, Muellheim, Germany), with a step size of 0.5 nm, over a wavelength range of 200–350 nm on a Jasco J-1100 spectropolarimeter (Jasco GmbH, Pfungstadt, Germany). Additionally, CD spectroscopy was employed to assess the conformational integrity of the purified nsP2^pro^ enzyme post-purification. The protease was diluted to a final concentration of 5 µM in a 10 mM sodium phosphate buffer (2.39 mM NaH₂PO₄, 7.6 mM Na₂HPO₄, pH 7.5). Measurements were conducted at 18 °C using a 1 cm pathlength cuvette across the wavelength range of 190–260 nm, with seven replicate scans to ensure data reliability. For data processing, baseline spectra, averaged from multiple measurements, were subtracted from the corresponding sample spectra to yield corrected readings. The resulting data were expressed as molar ellipticity ([θ]), calculated using Eq. (2):

[θ]λ=θ/(c*l*n)

Where θ represents the ellipticity measured at the wavelength λ (in degrees), c is the protein concentration (mol/L), l is the cell path length (cm), and n is the number of residues in the protein.

### 2.7 NMR spectroscopy

The ^15^N-labeled nsP2^pro^ samples were prepared in 20 mM bis-Tris propane (pH 7.5) with a 93%/7% (v/v) H_2_O/D_2_O mixture at a final protein concentration of 45 μM. The nsP2^pro^-ssDNA (5′-CGTCGCTATA-3′) complex was formed at a 1:1.1 protein:DNA molar ratio. nsP2^pro^ was also prepared in the presence of 2 mM Tris(2-carboxyethyl)phosphine hydrochloride (TCEP) to reduce cysteines. NMR spectra were acquired at 25 °C on a Bruker Avance III HD 700 MHz spectrometer equipped with a cryogenically cooled *z*-gradient triple resonance probe. Proton chemical shifts were referenced to 2,2-dimethyl-2-silapentane-5-sulfonate and ^15^N chemical shifts were indirectly referenced according to the ratio given by Wishart et al. [37]. Data sets were processed with NMRPipe and analyzed using CcpNMR Analysis [38,39].

### 2.8 *In silico* analysis

Models of CHIKV nsP2^pro^ and in complex with nucleic acids were generated using the AlphaFold 3 web server [40]. The structure of CHIKV nsP2^pro^ was retrieved from the PDB (code: 3TRK) and compared with the AlphaFold model (Fig. S2). To predict amino acids with the potential to interact with nucleic acids, the nsP2^pro^ alpha fold model was investigated with the PROBind webserver (https://www.csuligroup.com/PROBind/home) [41].

### 2.9 Molecular dynamics simulations

The initial poses for CHIKV nsP2^pro^-RNA and CHIKV nsP2^pro^-DNA complexes were chosen based on energy evaluations and visual inspection of the docking results obtained from the AlphaFold 3 web server [40]. Molecular dynamics (MD) simulations were performed using the AMBER24 suite [42]. The FF19SB force field [43] was used to describe protein atomic interactions, while OL21 parameters [44] were applied for DNA and OL3 parameters [45] for RNA. The protonation states of the protein were assigned using the H++ web server [46] at pH 7.5. All complexes were neutralized with Na or Cl ⁺ ions and placed in an octahedral box of TIP3P water extending 10 Å from the solute atoms. ⁻ Energy minimization was conducted in two stages: initially with positional restraints on the protein RNA and protein-DNA complexes using a constant force of 10.0 kcal/mol·Å² for 5000 steps of steepest descent followed by 5000 steps of conjugate gradient minimization. This was followed by a second minimization stage without restraints for 10,000 steps. The systems were then heated from 0 to 310 K under a constant number of atoms, volume, and temperature (NVT ensemble), with restraints of 10.0 kcal/mol·Å² maintained on the complexes. Equilibration was carried out in six steps, each lasting 500 ps, under a constant number of atoms, pressure, and temperature (NPT ensemble), with the restraint force gradually reduced from 10.0 kcal/mol·Å² to zero. The production phase was run for 200 ns using a 2 fs time step. The temperature (310 K) and pressure (1 atm) were controlled via Langevin dynamics. Long-range electrostatic interactions were treated using the Particle Mesh Ewald (PME) method [47], and a cut off distance of 10 Å was applied for van der Waals interactions.

### 2.10 Molecular dynamics analyses and energy interaction calculations

Analyses of the MD simulations were performed using CPPTRAJ [48], part of the AmberTools25 [42] package. Equilibration and convergence were assessed by root mean square deviation (RMSD), and protein flexibility was evaluated through root mean square fluctuation (RMSF). The radius of gyration and solvent-accessible surface area (SASA) were calculated for the protein to assess structural changes. Hydrogen bonds between the RNA/DNA and the protein were also computed to evaluate interaction stability. The Molecular Mechanics/Generalized Born Surface Area (MM/GBSA) method was used to calculate binding energies, based on snapshots from the last 100 ns of the production run, corresponding to the stable regime. All ions and water molecules were stripped from the systems prior to energy calculations. The POCASA 1.1 (POcket-CAvity Search Application) webserver was used to determine the change of the volume in the active site pocket after RNA and DNA binding [49].

### 2.11 *In silico* alanine scanning

Computational alanine scanning was performed using the MM/GBSA approach implemented in the MMPBSA.py module of AmberTools 25 [42] to identify residues involved in protein–DNA and protein–RNA recognition. The residues were individually mutated to alanine (R678A, R746A, S747A, R749A, N768A, F769A, D770A, N771A, R773A, and R774A), and their contributions to nucleic acid binding were evaluated using a single-trajectory protocol. The calculations were performed on snapshots extracted from the last 100 ns of the molecular dynamics trajectories, corresponding to the equilibrated portion of the simulations. Binding free energies were estimated using molecular mechanics energies combined with the generalized Born implicit solvent model, and the effect of each mutation was quantified as the change in binding free energy upon alanine substitution. A duplicate was performed accordingly.

### 2.12 Statistical analysis

To assess the significance level (p-value), statistical analyses were performed using GraphPad Prism (version 5.0). One-way ANOVA followed by Tukey's post hoc test was used to evaluate the differences between the groups and the control. Significance levels are indicated as follows: p < 0.05 (*), p < 0.01 (**), and p < 0.001 (***).

## 3 Results

### 3.1 DNA aptamer selection

Aptamers are short, single-stranded DNA or RNA molecules that fold into defined secondary and tertiary structures, allowing them to bind selectively and with high affinity to specific target molecules [50,51]. To isolate DNA aptamers capable of binding the CHIKV nsP2^pro^, a High-Throughput Sequencing–Fluorescent Ligand Interaction Profiling (HiTS-FLIP) approach incorporating a primer-blocking strategy was employed. This method facilitated the direct selection of high-affinity aptamers from a large pool of randomized DNA sequences. Based on their strong binding affinity to the nsP2^pro^, ten candidate aptamers were selected for further analysis. The sequences and corresponding K_D_ values of the top ten aptamers, named DAC1–DAC10 (DNA against Chikungunya), are listed in Table 2, while the K_D_ fitting data are shown in Fig. S3.

**Table 2:** Sequences and properties of the selected DNA aptamers (DAC1–DAC10).

Aptamer	Sequence (5′-3′)	K_D_ [nM]^1^	Tm [°C]^2^
DAC1	CTTTTATCAACTCACACTTTTCGTAAGTTTCTTCTTAAATCGCCGCACTT	37 ± 19	64.4
DAC2	CCGAACTTTTCTCTTTTAGATTGGAATCTAGGTCAATGTTGTATTTAATC	1 ± 0.7	61.6
DAC3	CAAACATTTCTGCGAATATTTCTCCCGATACCTGTTGTGTTAAACCAGCC	3 ± 1.5	66.1
DAC4	TTTTAGACCACAGTAACATCCTGAACATGGCACTCGGCGCGATTTTCCTT	8 ± 2	68.9
DAC5	ATATAGCAGTTGGGCTCATTTGTCCACCACTGAAAGCCCGAAAACCCGTT	8 ± 2	69.9
DAC6	ACACTGTACCAGCGTTTATTTTCTATCCGTATTTTTAGGTTCTTTATTTC	14 ± 6	62.4
DAC7	ATCTCCTACCCGACGTGACTATACTATCTGTCGTATTCCGTCTTCTGATT	0.5 ± 0.4	66.1
DAC8	GGCCTCAAGCACGCCTTCATAACTTCTGCTTACCTAGATTGGATATTAGT	1 ± 0.6	66.7
DAC9	CATTGATTTATCACTTTATAGTATTAATCACTGGGATCAATCGCTCCTGA	3 ± 1	62.6
DAC10	AATTCACCCGGTCCCGCGTGAGGTTCTATCTAACTTAGCGACAGAGACCT	9 ± 4	70.1

1 Apparent dissociation constants (K_D_) for DNA aptamers were calculated based on fluorescence intensities obtained from HiTS-FLIP experiments by Hill-fits.

2 Melting temperatures (T_m_) of DNA aptamers were calculated using the OligoAnalyzer Tool from Integrated DNA Technologies, Inc.

### 3.2 Secondary structure prediction of the nucleic acids

To evaluate the secondary structure of the selected DNA aptamers targeting CHIKV nsP2^pro^, the DINAMelt Server – Quikfold web tool was utilized [36]. This tool predicts folding conformations based on thermodynamic parameters (Figs. S4 and S5). The structural predictions revealed that all DNA aptamers could adopt secondary structures characterized by hairpin formations, although the lengths of the stems and sizes of the loops varied among the different sequences. To experimentally validate these computational predictions, CD spectroscopy was performed to assess the secondary structure content of the aptamers (Figs. S6–S8). CD spectroscopy results showed similar structural features under both ddH₂O and 1 × PBS buffer conditions, with two positive peaks around 280 nm and 220 nm, and a negative peak near 245 nm. In addition to the DNA aptamers, two single-stranded RNA molecules RAC1 (5 nucleotides) and RAC2 (10 nucleotides), where “RAC” denotes RNA Against Chikungunya (Table S1), were analyzed by CD spectroscopy to evaluate their secondary structure characteristics. RAC1 exhibited a broad spectrum with low ellipticity, showing a weak positive peak around 270–280 nm, suggesting minimal or disordered secondary structure. In contrast, RAC2 displayed a more defined CD spectrum, with a clear positive peak at 270–280 nm and a distinct negative peak at around 240 nm, indicating the formation of a more ordered secondary structure (Fig. S8).

### 3.3 Impact of DNA aptamers on CHIKV nsP2^pro^ enzymatic activity

**Fig. 1:**
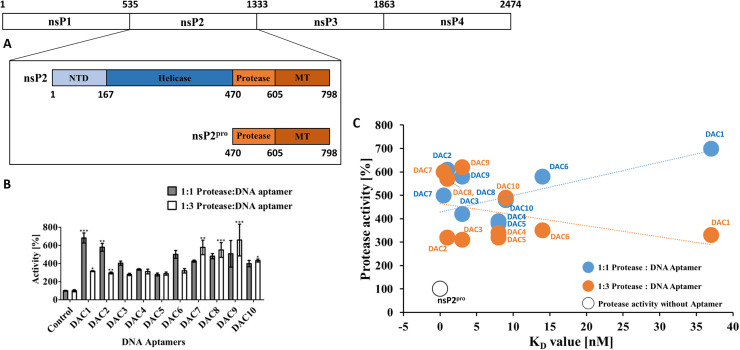
*Enzymatic activity of* nsP2^pro^*in the presence of DNA aptamers (DAC1–DAC10) at different molar ratios.* (A) Schematic representation of the nsP2^pro^ construct comprising the amino acid residues used in this study.. (B) A primary activity assay of DNA aptamers was performed in 1 × PBS at two different molar ratios: 1:1 and 1:3. The protease concentration was 10 µM, and the substrate concentration was maintained at 9 µM. The protease was mixed with DNA aptamers at 1:1 (10 µM, gray bars) and 1:3 (30 µM, white bars) molar ratios. Data from three independent experiments (n = 3) are presented as mean ± standard deviation (SD). Statistical significance was evaluated using one-way ANOVA followed by Tukey’s post hoc test. Asterisks indicate values that differ significantly from the respective control group. The significance levels are defined as follows: p < 0.05 (*), p < 0.01 (**) and p < 0.01 (***). If no asterisk is shown, the difference was not statistically significant (p > 0.05). (C) Relationship between nsP2pro activation by aptamers and their K_D_ values. The two tested aptamer concentrations are shown and the protease activity without aptamer.

To investigate the impact of nucleic acids on the enzymatic activity of nsP2^pro^ (Residues 470–798 of nsp2, Fig. 1A), a primary activity assay was conducted in 1x PBS buffer using DNA aptamers targeting CHIKV nsP2^pro^ (DAC1–DAC10). The aptamers were mixed with 10 µM nsP2^pro^ at two molar ratios: 1:1 (10 µM) and 1:3 (30 µM). Fluorescence intensities (excitation at 340 nm, emission at 490 nm) were recorded every 30 s over a 30-min. As shown in Fig. 1B, all tested DNA aptamers significantly enhanced nsP2^pro^ activity. Among the ten aptamers variants, DAC1 exhibited the most pronounced effect, increasing enzymatic activity by approximately 600%. DAC2, DAC6, DAC8, and DAC9 induced up to 500% enhancement, while DAC3, DAC4, and DAC5 demonstrated moderate levels of activation (approximately 300–400%), suggesting differences in their ability to promote protease activity. The results also revealed that nsP2^pro^ activity was influenced by the aptamer-to-protease molar ratio. For most DAC variants, increasing the aptamer concentration from 10 µM to 30 µM did not further enhance activity. In fact, for DAC1, DAC2, DAC3, DAC4, and DAC6, higher concentrations led to a reduction in enzymatic activity, suggesting that higher aptamer concentrations do not lead to the higher activity rate in these cases. In contrast, DAC7, DAC8, DAC9, and DAC10 showed a slight increase in activity at the 1:3 ratio compared to 1:1, indicating only minimal additional enhancement.

Blotting the aptamer K_D_ values against the corresponding enzymatic activities, demonstrated an inverse relationship between binding affinity and activation potential, with aptamers exhibiting higher K_D_ values (weaker binding) (DAC1 and DAC6) generally promoting stronger activation of nsP2^pro^, compared to the stronger binding aptamers (Fig. 1C).

### 3.4 Impact of random ssDNA, dsDNA, ssRNA, and nucleotides on the enzymatic activity of CHIKV nsP2^pro^

**Fig. 2:**
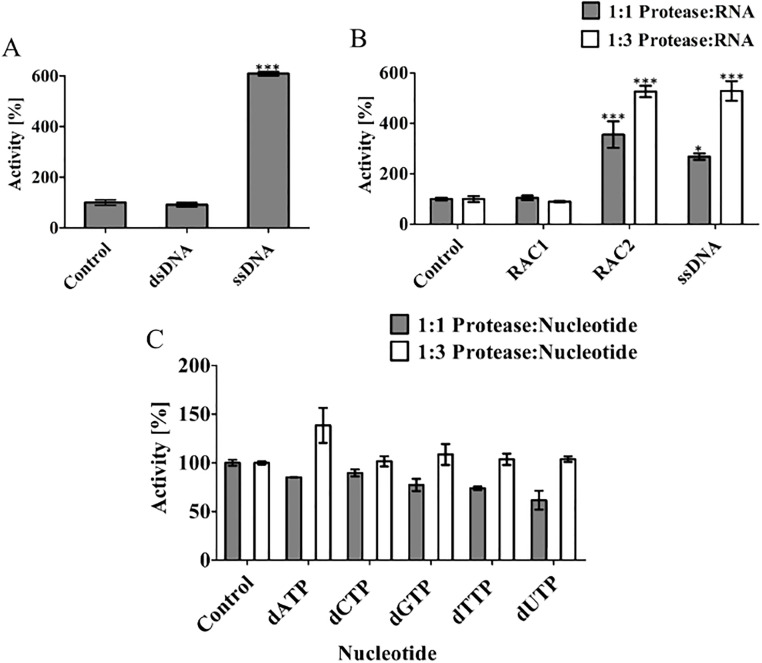
Influence of nucleic acids and nucleotides on nsP2^pro^ enzymatic activity. (A) Effect of random ssDNA and dsDNA on nsP2^pro^ activity. (A) Primary assay was conducted to assess the influence of random ssDNA (5′- TGACCATGGAGCCTGCCGTCTACTTCAAG-3′) and dsDNA on the enzymatic activity of nsP2^pro^. (B) Enzymatic activity of nsP2^pro^ in the presence of ssRNAs (RAC1–2) and ssDNA (5′-CGTCGCTATA-3′) corresponding to RAC2, tested at 1:1 (10 µM, gray bars) and 1:3 (30 µM, white bars) molar ratios. (C) Enzymatic activity of nsP2^pro^ in the presence of single nucleotides. Assays were performed in 1 × PBS at two molar ratios: 1:1 (10 µM, gray bars) and 1:3 (30 µM, white bars). In all experiments, the protease concentration was 10 µM and the substrate concentration was maintained at 9 µM. Data are shown as mean ± standard deviation (SD) from three independent experiments (n = 3). Statistical significance was evaluated using one-way ANOVA followed by Tukey’s post hoc test. Asterisks indicate significant differences compared to the control group, with thresholds defined as: p < 0.05 (*), p < 0.01 (**), and p < 0.001 (***). If no asterisk is shown, the difference was not statistically significant (p > 0.05).

In the previous section, we showed that selected DNA aptamers enhance the enzymatic activity of CHIKV nsP2^pro^. To evaluate the specificity of this effect, random single-stranded DNA (ssDNA) was used as a control. Interestingly, random ssDNA at a 1:1 molar ratio with the protease, also enhanced enzymatic activity, increasing it by up to 600% (Fig. 2A). To further investigate the effect of random DNA on nsP2^pro^ activity, the potential of random dsDNA, isolated from TgM83^+/−^ mice, was also examined. In contrast to ssDNA, dsDNA did not induce any detectable increase in nsP2^pro^ activity (Fig. 2A), indicating that the stimulatory effect is likely dependent on the single-stranded conformation of the nucleic acid.

Given that CHIKV is an RNA virus that replicates in the cytoplasm of host cells, it was of interest to evaluate whether RNA can influence the activity of nsP2^pro^. To investigate this, two ssRNA oligonucleotides, 5-mer and 10-mer sequences, randomly derived from the CHIKV genome were tested. These were designated RAC1 (5 nucleotides) and RAC2 (10 nucleotides) (Table S1). The primary activity assay, conducted in 1x PBS buffer, showed that the shorter ssRNA, RAC1, did not enhance nsP2^pro^ enzymatic activity at either the 1:1 or 1:3 molar ratios (Fig. 2B). However, RAC2 significantly increased protease activity. At 10 µM, activity increased to around 300%, and at 30 µM, it rose to approximately 500% compared to the control (Fig. 2B). In addition, we tested a ssDNA oligonucleotide with the same sequence as RAC2. Interestingly, this ssDNA also enhanced nsP2^pro^ enzymatic activity, increasing it by approximately 260% at the 1:1 molar ratio and around 500% at the 1:3 molar ratio, similar to the effect observed with RAC2 (Fig. 2B). To investigate whether individual nucleotides could enhance nsP2^pro^ activity, a primary activity assay was conducted under the same experimental conditions. Nucleotides were tested at concentrations of 10 µM and 30 µM. At the lower concentration (10 µM), no significant increase in protease activity was observed. However, at 30 µM, a modest increase in protease activity was detected (Fig. 2C). Specifically, dATP at 30 µM induced a modest increase of approximately 38%, which is substantially lower than the activity observed in the presence of the ssDNA or ssRNA.

### 3.5 Impact of buffer composition on the activity of CHIKV nsP2^pro^ by DNA aptamer and ssRNA

**Fig. 3:**
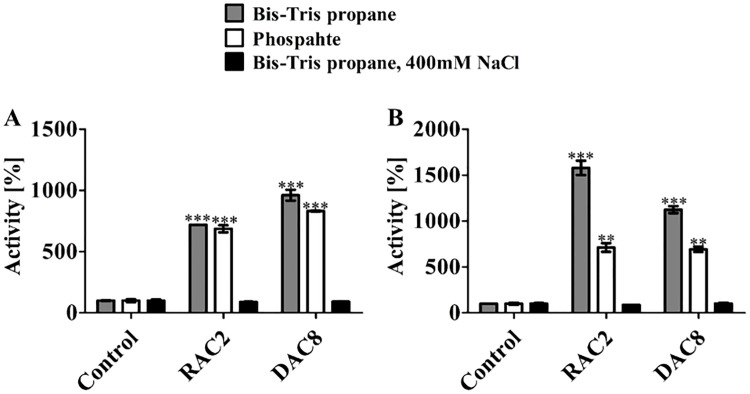
Effect of buffer composition on the activity of nsP2^pro^ by nucleic acids. The activity of nsP2^pro^ by DAC8 (a DNA aptamer) and RAC2 (ssRNA) was assessed in the presence of three different buffer conditions: (1) 20 mM Bis-Tris propane, pH 7.5, (2) 20 mM phosphate buffer, pH 7.5, and (3) 20 mM Bis-Tris propane, 400 mM NaCl, pH 7.5. Nucleic acids were tested at molar ratios of 1:1 (A) and 1:3 (B) with nsP2^pro^ (10 µM). The substrate concentration was kept at 9 µM. Data are presented as the mean ± SD from three independent experiments (n = 3). Statistical significance was evaluated using one-way ANOVA followed by Tukey’s post hoc test. Asterisks indicate significant differences compared to the control group, with thresholds defined as: p < 0.05 (*), p < 0.01 (**), and p < 0.001 (***). If no asterisk is shown, the difference was not statistically significant (p > 0.05).

To assess the effect of different buffers on nsP2^pro^ activity in the presence of nucleic acids, the ability of DAC8 (a representative DNA aptamer) and RAC2 (a 10-mer ssRNA) to enhance protease activity was evaluated under various buffer conditions. Assays were conducted using 10 µM nsP2^pro^ and two molar ratios of protease-to-nucleic acids: 1:1 and 1:3. Three buffer systems were tested: 20 mM Bis-Tris propane (pH 7.5), 20 mM phosphate buffer (pH 7.5), and 20 mM Bis-Tris propane supplemented with 400 mM NaCl (pH 7.5). As shown in Fig. 3A–B, both DAC8 and RAC2 enhanced protease activity in Bis-Tris propane and phosphate buffers at both molar ratios. Notably, DAC8 induced a more pronounced increase in activity than RAC2 at the 1:1 ratio. However, at the 1:3 ratio, RAC2 exhibited greater activation than DAC8 in Bis-Tris propane buffer, while in phosphate buffer, both nucleic acids produced similar effects. In contrast, under high-salt conditions (400 mM NaCl), neither DAC8 nor RAC2 was able to stimulate nsP2^pro^ activity at either molar ratio. These findings suggest that elevated ionic strength inhibits nucleic acid-mediated enhancement of protease activity and underscore the critical role of buffer composition in modulating nsP2^pro^ enzymatic function.

### 3.6 Interaction of nsP2^pro^ with ssDNA leads to conformational and dynamic changes

**Fig. 4:**
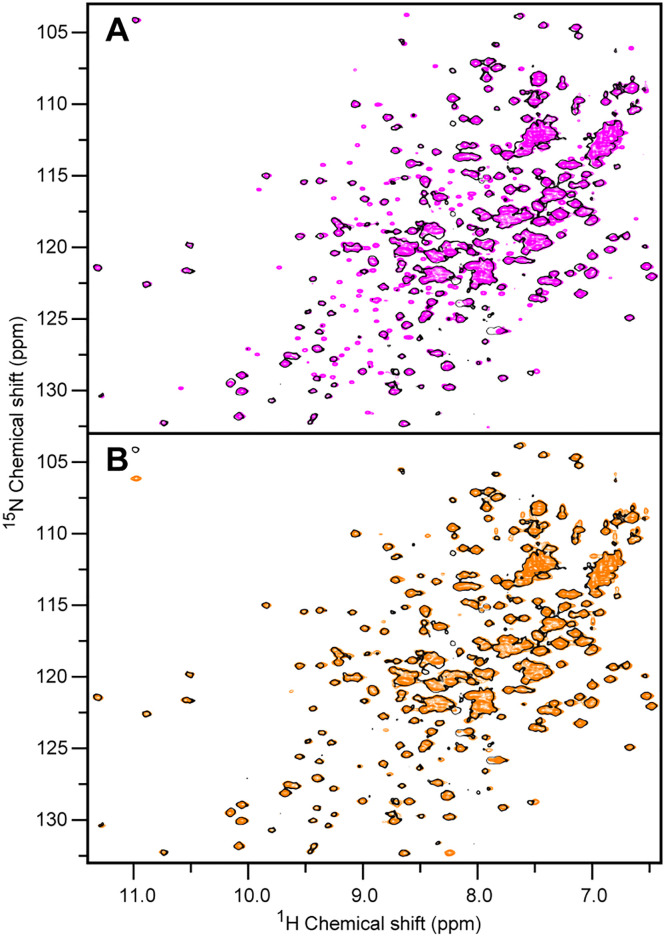
Changes in dynamics and conformation of nsP2^pro^ upon ssDNA binding and in the presence of the reducing agent TCEP. Overlay of 2D ^1^H–^15^N HSQC spectra of ^15^N-labeled nsP2^pro^ in the (A) presence of ssDNA and (B) presence of TCEP. (A) The spectrum of nsP2^pro^ is shown in pink, whereas the spectrum of nsP2^pro^ in the presence of ssDNA is shown in black drawn with seven contour levels. A set of ∼40 cross peaks are absent in the nsP2^pro^–ssDNA spectrum, consistent with localized conformational exchange and/or altered dynamics affecting a subset of residues upon ssDNA interaction. (B) Mild reduction with TCEP yields a 2D ^1^H–^15^N HSQC spectrum (orange) that is essentially identical to the DNA-bound state spectrum (black), suggesting that a redox-sensitive conformational equilibrium biases nsP2^pro^ toward the ssDNA-bound state conformation.

NMR spectroscopy was used to examine the non-specific interaction between nsP2^pro^ and ssDNA. In the 2D ^1^H–^15^N HSQC, addition of ssDNA caused the disappearance of a subset of cross peaks, whereas the remainder of the spectrum overlaid closely with the apo state spectrum (Fig. 4A). This behavior is consistent with localized conformational and/or dynamic changes affecting a subset of residues upon ssDNA interaction, rather than a global change to the protein. Interestingly, mild reduction with TCEP produced a 2D ^1^H–^15^N HSQC spectrum that closely resembles the ssDNA-bound state spectrum (Fig. 4B), suggesting that a redox-sensitive conformational equilibrium is responsible for these observations, with cysteine reduction biasing the ensemble toward the ssDNA-bound state conformation. Residue-specific resonance assignments, which would be required for detailed structural interpretation of these spectral changes, are beyond the scope of the present study. Further research clarifying the mechanism responsible for these NMR-based observations may be addressed using relaxation dispersion and related NMR methods [52].

### 3.7 Possible binding mode of nucleic acids with CHIKV nsP2^pro^

**Fig. 5:**
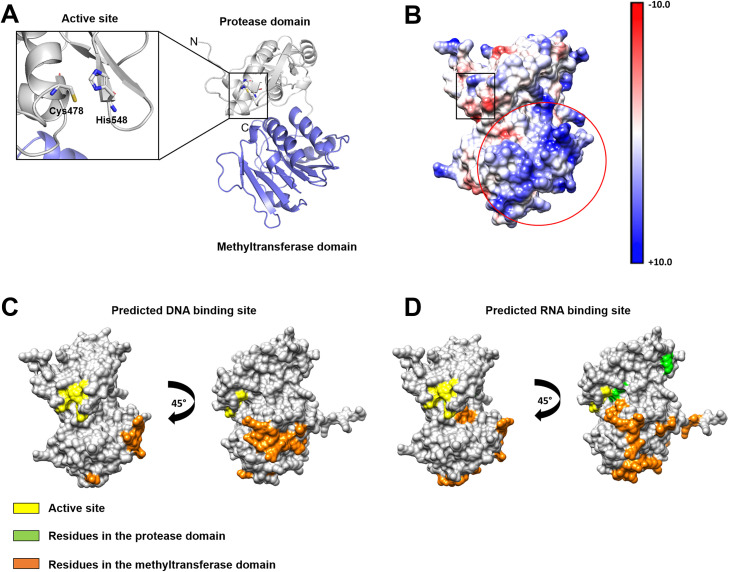
Investigation of the nsP2^pro^ AlphaFold model with regard to possible nucleic acid binding regions. A structural comparison between the crystal structure of the protein (PDB code: 3TRK) and the generated AlphaFold model is shown in S2 Fig. (A) nsP2^pro^ is shown in ribbon view, the papain-like cysteine protease is colored in grey and the Ftsj methyltransferase (MTase)-like domain in blue. The black box labels the position of the active site residues Cys478 and His548. (B) Coulombic surface of nsP2^pro^, the red circle labels a strongly positively charged area at the MTase domain, the black box labels the position of the active site residues. The online tool PROBind was used to predict amino acids that can take part in interactions with DNA and RNA. The protein is shown in surface view and the predicted amino acids are labeled in orange (MTase domain) and green (Protease domain). The position of the active site is labeled in yellow. (C) Predicted DNA binding site. (D) Predicted RNA binding site.

CHIKV nsP2^pro^ consists of an N-terminal papain-like protease domain, directly followed by a Ftsj MTase-like domain (Fig. 5A). The surface of the Mtase-like domain possesses clusters of positive charges, located near the protease active site loop carrying His548 (Fig. 5B), which may propagate the binding of the negative charged phosphate backbone of nucleic acids.

The online tool PROBind [41] was used to predict amino acids that are involved in DNA and RNA binding (Fig. 5C-D). Thereby, 14 residues were predicted to interact with DNA, located mainly at a surface cluster in the Mtase domain (Fig. 5C). 23 residues were predicted to interact with RNA, where 16 residues are located at the Mtase domain and 7 residues at the active site loop carrying His548 on the protease domain. Some areas seem to overlap regarding the DNA and RNA interactions, the predicted amino acids are shown in the nsP2^pro^ sequence at Fig. S9.

Structural models of complexes between CHIKV nsP2^pro^ and ssDNA (5′-CGTCGCTATA-3′) and RAC2 (5′-CGUCGCUAUA-3′) were generated using the AlphaFold 3 web server [40]. Subsequent molecular dynamics (MD) simulations were performed to investigate the possible binding interface between the protease and the nucleic acids. AlphaFold typically generates five predicted protein structures per run, and these are ranked based on their confidence scores. The structure with the lowest rank (typically rank 0) is generally considered the best prediction [40]. The five predicted structures for CHIKV nsP2^pro^-DNA and RNA showed a consistent localization at the Mtase domain and the predicted binding regions for DNA and RNA (Fig. 5C and D and Fig. S10). For molecular dynamics simulation rank 0 was chosen as the starting pose. A duplicate of 200 ns was performed and the flexibility of the protease and nucleic acids (RNA and DNA) in complex through MD simulation was monitored by calculating the RMSD, RMSF, RoG, and the surface area (Figs. S11 and S12). We noticed moderate deviations in the RMSD, RMSF, Rog and surface area values for all backbone atoms regarding the starting structure along the 200 ns trajectories, in two independent MD simulation runs.

**Fig. 6:**
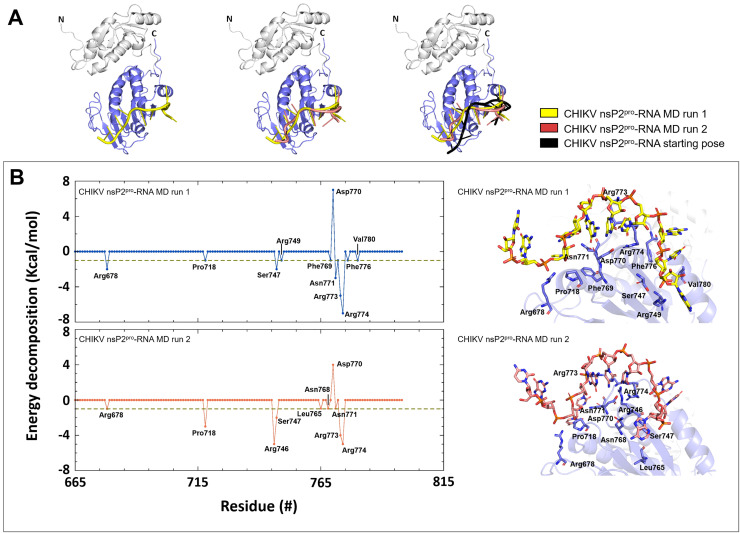
CHIKV nsP2^pro^-RNA (5′-CGUCGCUAUA-3′) complex after 200 ns MD simulation. (A) Coordination of RNA at the nsP2^pro^ Mtase domain and overlay of the MD simulation replicates 1 (Yellow) and 2 (Salmon) and the initial RNA position before MD simulation (Black). (B) Amino acids contributing in the CHIKV nsP2^pro^–RNA interaction. Decomposition energy of the amino acids involved in the interaction with RNA based on the MD simulations. A detailed view on the protease-RNA complex is shown for both MD runs, the involved amino acids in the interaction with the RNA are represented as sticks.

**Fig. 7:**
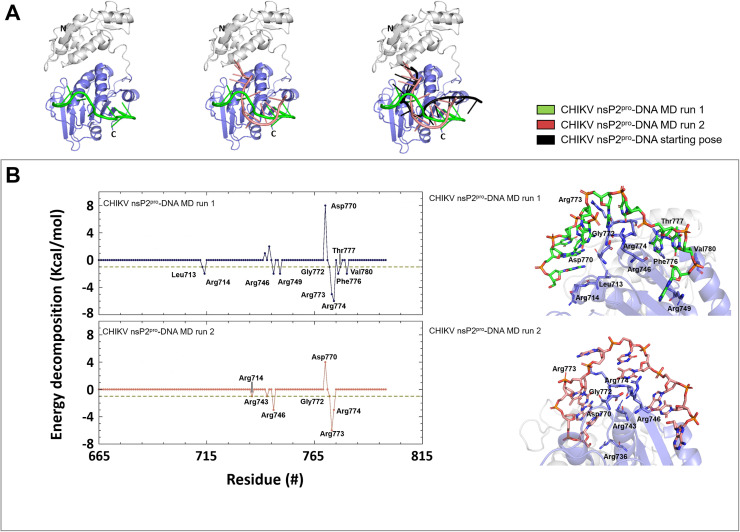
CHIKV nsP2^pro^-DNA (5′-CGTCGCTATA-3′) complex after 200 ns MD simulation. (A) Coordination of DNA at the nsP2^pro^ Mtase domain and overlay of the MD simulation replicates 1 (Green) and 2 (Salmon) and the initial RNA position before MD simulation (Black). (B) Amino acids contributing in the CHIKV nsP2^pro^–DNA interaction. Decomposition energy of the amino acids involved in the interaction with DNA based on the MD simulations. A detailed view on the protease-DNA complex is shown for both MD runs, the involved amino acids in the interaction with the DNA are represented as sticks.

The representative structure calculated for the trajectory of the most stable RNA and DNA poses in complex with CHIKV nsP2^pro^, are depicted in Figs. 5A and 6A. As can be observed the final nucleic acid binding mode and position diverges slightly compared to the second MD run and the initial starting position (Figs. 6A and 7A), which is also deduced by the RNA and DNA RMSD profile (Figs. S11 and S12).

The analysis of the amino acid residues involved in the interaction with RNA within the two independent MD simulation runs show a similar interaction pattern (Fig. 6B). Thereby Arg773 and Arg774 show the strongest energy decomposition. Interestingly, the adjacent residue Asp775 seems to have a repulsive effect on the RNA. The same binding pattern can be observed for the nsP2^pro^ interaction with DNA (Fig. 7B).

**Fig. 8:**
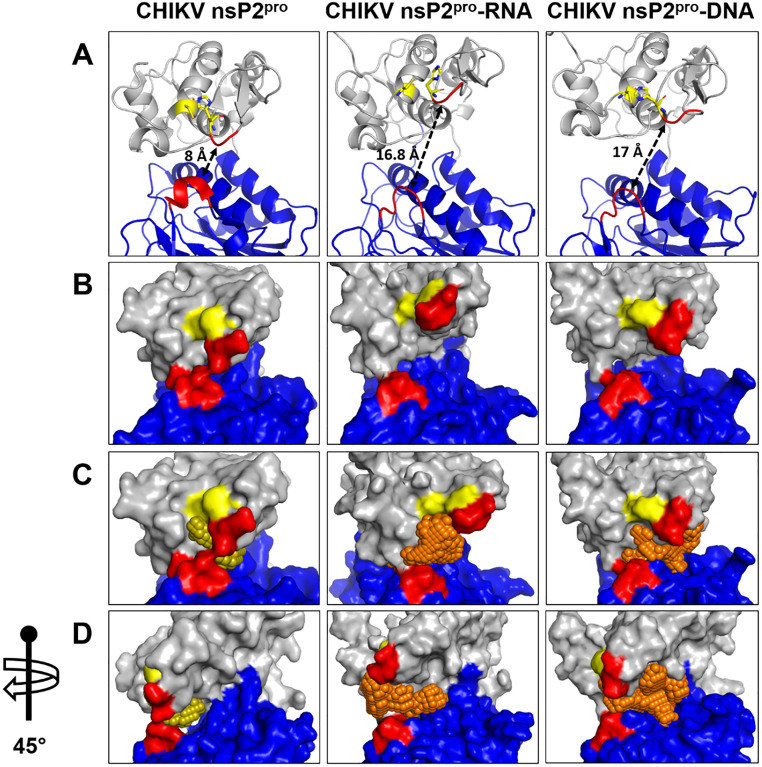
CHIKV nsP2^pro^ active site movement after MD simulations with and without nucleic acids. The active site region is shown in ribbon view and in surface view. The protease domain is colored in grey, the Mtase domain in blue, the catalytical residues (Cys478 and His548) in yellow, the protease active site loop and the Mtase loop _667_NLELGL_672_ are colored in red and the volume in orange. Row (A) show the distance in Å between the protease active site loop carrying His548 and the Mtase loop. From left to the right: CHIKV nsP2^pro^, 8 Å. CHIKV nsP2^pro^-RNA, 16.8 Å and CHIKV nsP2^pro^-DNA 17.0 Å. Row (B) active site region in surface view. From left to right: CHIKV nsP2^pro^, CHIKV nsP2^pro^-RNA and CHIKV nsP2^pro^-DNA. Rows (C) and (D) (rotated at 45°) active site region in surface view with determined volume. From left to right: CHIKV nsP2^pro^, CHIKV nsP2^pro^-RNA and CHIKV nsP2^pro^-DNA.

The amount of amino acids involved in the interaction with the tested nucleic acids are different. In the interaction with RNA 12 and 11 amino acids are involved in both MD runs and for DNA 11 and 7. The amino acids are located exclusively in the Mtase domain and no residues are located in the protease domain as predicted by PROBind (Table S2). Furthermore, the number of formed hydrogen bonds over the simulation time between the protease RNA (4 hydrogen bonds in both replicates) was higher in comparison with DNA (1 and 0.7 hydrogen bond, respectively) (Fig. S13). Moreover, the MD simulations demonstrated a conformational change of the active site loop were His548 is located, in both replica for the nsP2^pro^ complex with RNA and DNA, an opening process of the active site can be observed compared to the nsP2^pro^ crystal structure (PDB code: 3TRK) (Fig. 8A and B and Fig. S14). In the closed state of the active site, the active site loop and the Mtase loop _667_NLELG_672_ have a distance of around 8.0 Å. After MD simulations with RNA and DNA the distance between this both regions increased to around 16.8 to 17.0 Å (Fig. 8A).

The Volume of the nsP2^pro^ active site pocket increases after the interaction with nucleic acids (Fig. 8C and B). To determine the volume of the active site pocket, the pocasa 1.1 webserver [45] was used. The results are shown in Table 3.

**Table 3:** Determined volume of the nsP2^pro^ active site pocket, with and without nucleic acids. Numbers in red are the volume of the pockets, shown in Fig. 6C and D.

Protein/complex	Volume [Å^3^]	Conformation
nsP2^pro^	155	closed
nsP2^pro^-RNA (run1)	**926**	open
nsP2^pro^-RNA (run2)	867	open
nsP2^pro^-DNA (run1)	**841**	open
nsP2^pro^-DNA (run2)	701	open

After binding RNA or DNA, respectively, the conformation of the active site changes to a more open state and the Volume increase about the 4 to 5 times.

**Fig. 9:**
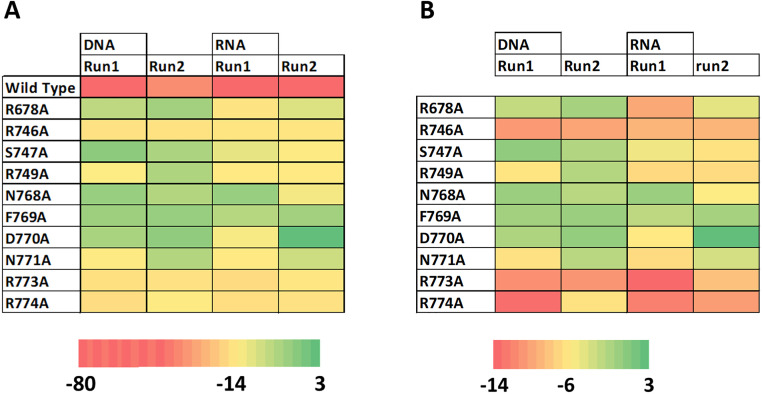
Heat map representation of computational alanine scanning results of the putative CHIKV nsP2pro nucleic acid binding region. Duplicates of the alanine scanning are shown and the binding energy is represented in kcal/mol. **(A)** Alanine scanning and respresentative energy values of residues R678, R746, S747, R749, N768, F769, D770, N771, R773, and R774, compared with the nsP2^pro^ wildtype. (B) Alanine scanning and respresentative energy values of residues R678, R746, S747, R749, N768, F769, D770, N771, R773, and R774. The binding energy of all mutants are shown in Table S3.

To systematically identify residue-specific interaction hotspots within the proposed nucleic acid-binding region of CHIKV nsP2^pro^, an *in silico* alanine scanning analysis was conducted. Individual alanine substitutions were introduced at residues R678, R746, S747, R749, N768, F769, D770, N771, R773, and R774. The effects of these substitutions on the predicted interactions with DNA and RNA were subsequently evaluated to assess their relative contributions to nucleic acid recognition. The resulting interaction profiles are depicted as a heat map in Fig. 9.

This *in silico* approach identified several residues that make major contributions to nucleic acid recognition by CHIKV nsP2^pro^. Alanine substitution significantly perturbed the predicted binding energetics, with binding energies changing from approximately −80 kcal/mol for the wild-type protein to approximately −14 kcal/mol for the R773A and R774A variants, while D770A displayed the largest change (approximately 3 kcal/mol; Fig. 9A). Comparative analysis of individual substitutions further demonstrated that R678 contributes more substantially to nucleic acid binding than R746, R749, R773, or R774, indicating a dominant role for this residue within the positively charged binding surface. Among the arginine residues investigated, their relative contributions followed the order R678 > R749 > R746 > R774 > R773. Remarkably, alanine substitution of N768 and F769 resulted in the greatest loss of binding. Moreover, nucleic acid-dependent effects were evident, as R678A and S747A preferentially impaired DNA binding relative to RNA binding. In contrast, mutations N768A and F769A severely compromised the nucleic acid-binding capacity of this region irrespective of substrate identity.

## 4 Discussion

The Chikungunya virus (CHIKV) is a rapidly emerging arthropod-borne pathogen whose increasing global distribution is related to climate and environmental changes. Central to CHIKV replication is its nonstructural protein 2 (nsP2), a multifunctional enzyme that plays an important role as a viral protease (nsP2^pro^) by mediating the cleavage of the viral polyprotein. Several studies have reported that interactions between viral proteins and nucleic acids are pivotal for mediating key aspects of the viral life cycle. For example, the Rep protein of adeno-associated virus type 2 binds single-stranded DNA (ssDNA), a process that facilitates viral replication [53] and HIV-1 protease has been shown to bind viral RNA, potentially augmenting its catalytic activity [27]. In other cases, the binding of viral RNA to proteases such as those of the Foamy virus [23] and the 3C^pro^ protease of poliovirus [29] has been reported to activate protease function. Not only RNA but also DNA can mediate protease activation. For instance, host DNA has been shown to activate the 3C^pro^ of Seneca Valley virus [30], highlighting the potential role of host nucleic acids in modulating viral protein activity. Interestingly, nucleic acid interactions do not always facilitate viral replication; in some cases, they can inhibit it. For example, RNA aptamers have been identified that bind to the NS3 protease of hepatitis C virus, inhibiting its function in polyprotein processing [26]. Similarly, RNA aptamers targeting HIV-1 PR have been reported to block its enzymatic activity [28]. In light of these findings, this study focused on CHIKV nsP2^pro^ to assess the impact of nucleic acid interactions on its protease activity. HiTS-FLIP was employed to identify ssDNA aptamers that bind to nsP2^pro^. Ten aptamers (designated DAC1–DAC10) were selected based on their binding affinity (Table 3) and were subsequently tested for their effect on protease activity. To evaluate the effect of these aptamers on nsP2^pro^ activity, primary activity assays were conducted. The results showed that all ssDNA aptamers had the potential to enhance the activity of the target protease (Fig. 1). Among the ten DAC variants, DAC1 exhibited the most pronounced effect, increasing enzymatic activity by 600% (Fig. 1). DAC2, DAC6, DAC8, and DAC9 also induced substantial increases, reaching up to 500%. In contrast, DAC3, DAC4, and DAC5 showed moderate enhancement at a 1:1 ratio relative to the protease concentration (10 µM). However, when the DNA concentration was increased to a 1:3 ratio, a reduction in protease activity was observed, with DAC1-induced activity decreasing to approximately 300% compared to the 1:1 condition. This reduction may be attributed to the elevated ssDNA concentration, which could promote protein precipitation and aggregation-a phenomenon reported for proteins such as tau and α-synuclein, where nucleic acids have been shown to induce aggregation [54]. To ensure that the observed activity increase was due to protease–nucleic acid interactions and not simply optical interference from the aptamers themselves, control experiments were conducted in the absence of substrate. Reactions were prepared with buffer alone, protease plus buffer, and protease with buffer and DNA aptamers, excluding the fluorogenic substrate. No detectable absorbance was observed in the lack of substrate, confirming that the increased signal in the complete assays was attributable to enzymatic activity, not background fluorescence or absorbance changes caused by the aptamers (Fig. S15). To determine whether the observed increase in nsP2^pro^ activity was due to specific binding by the selected ssDNA aptamers or could also result from non-specific interactions, a random ssDNA sequence was tested. Interestingly, as shown in Fig. 2A, the random ssDNA also enhanced protease activity, suggesting that the effect is not sequence-specific. To further explore this phenomenon, the effect of a random dsDNA, isolated from TgM83^+/−^ mice, was examined. In contrast to ssDNA, the random dsDNA did not cause any enhancement in protease activity compared to the control (Fig. 2A), suggesting that the stimulatory effect is dependent on the single-stranded nature of the nucleic acid. Since CHIKV is a ssRNA virus that replicates in the cytoplasm, it is likely that nsP2^pro^ naturally encounters ssRNA during infection. To evaluate whether ssRNA could similarly influence protease activity, two random ssRNA oligonucleotides were tested: a 5-mer (RAC1) and a 10-mer (RAC2), both derived from the CHIKV genome (S1 Table). Activity assays revealed that RAC1 did not enhance nsP2^pro^ activity, whereas RAC2 significantly increased activity, reaching approximately 600% (Fig. 2B). These findings suggest that ssRNA, like ssDNA, can enhance protease activity, but the effect is length-dependent and requires a minimum RNA length. To confirm the length dependency, an additional assay was performed using single nucleotides. At a 1:1 molar ratio with the protease, no change in activity was observed; however, at a 1:3 ratio, a slight increase was detected (Fig. 2C). This result aligns with the ssRNA experiments and further supports the conclusion that enhancement of nsP2^pro^ activity by nucleic acids requires a minimal oligonucleotide length. While the primary objective of this study was to determine whether nucleic acids can broadly modulate nsP2^pro^ activity, future investigations are needed to define the sequence-, length-, and structure-dependent determinants underlying this regulatory effect.

The influence of buffer composition on nucleic acid-mediated activation of nsP2^pro^ was systematically evaluated using two representative oligonucleotides: DAC8 (ssDNA aptamer) and RAC2 (ssRNA). Three buffer systems were tested: 20 mM Bis-Tris propane (pH 7.5), 20 mM phosphate buffer (pH 7.5), and 20 mM Bis-Tris propane supplemented with 400 mM NaCl (pH 7.5). As shown in Fig. 3A–B, both DAC8 and RAC2 significantly enhanced protease activity in Bis-Tris propane and phosphate buffers without added salt. At the 1:1 ratio, DAC8 induced a stronger activation compared to RAC2, while at the 1:3 ratio, RAC2 exhibited greater enhancement in Bis-Tris propane. In phosphate buffer, both oligonucleotides led to similar effects. However, in the presence of high salt (400 mM NaCl), the activation was completely abolished for both DAC8 and RAC2, irrespective of the molar ratio. These results indicate that the stimulatory interaction between nucleic acids and nsP2^pro^ is highly sensitive to ionic strength. The loss of activity in high-salt conditions suggests that electrostatic interactions play a critical role in facilitating protease activity. This observation aligns with findings from other studies, such as those involving the hsRosR protein, demonstrated that high salt concentrations can disrupt protein-nucleic acid binding by altering solvent properties or changing the electrostatic environment of the protein and DNA and the correlation in between aptamer affinity and pI value of target proteins [55,56]. Therefore, the effect of high salt on nsP2^pro^ activity likely stems from the disruption of protein- nucleic acids interactions, emphasizing the importance of buffer conditions for nucleic acid-mediated protease activation. To gain a better understanding of the structural basis of the DNA aptamers, the secondary structures of the selected DNA aptamers were predicted using the DINAMelt Server – Quikfold web tool [35]. The predictions revealed that all sequences adopted secondary structures characterized by hairpin formations, although the lengths of the stems and the sizes of the loops varied among the different aptamers (Figs. S4–S5). CD spectroscopy further confirmed the presence of these structured forms in both ddH₂O and 1 × PBS conditions. The spectra of all DNA aptamers exhibited two distinct positive peaks around 280 nm and 220 nm, along with a strong negative peak near 245 nm. A pattern consistent with the B-form DNA conformation [57] (Figs. S6–S8). These spectral features corroborate the computational predictions and indicate that all DNA aptamers retain stable secondary structures in solution. Furthermore, comparative CD analysis of these DNA aptamers with two ssRNA molecules (RAC1 and RAC2) revealed marked structural differences. RAC2 exhibited a spectral profile characteristic of the A-form RNA helix, including a moderate positive band near 270 nm, reflecting a well-structured RNA fold [58]. In contrast, RAC1 showed significantly diminished ellipticity across the spectrum, consistent with an unfolded or denatured state. This interpretation is in agreement with previous findings, where unfolded RNAs lack the defined CD spectral features associated with stable secondary structures [58] (Fig. S8). These findings suggest that the secondary structure of nucleic acids may influence their binding capacity to nsP2^pro^ and contribute to their ability to enhance enzymatic activity.

The results of the 2D ^1^H–^15^N HSQC experiments demonstrated that interaction of nsP2^pro^ with ssDNA caused disappearance of a subset of cross peaks in the NMR spectrum, whereas the rest of the spectrum remained nearly identical to that of the apo-form (Fig. 4A). These observations are consistent with two non-exclusive explanations. One possibility is a two-state behavior on the slow chemical-shift exchange timescale, in which a region of the apo-nsP2^pro^ samples two distinct, NMR-visible conformers and DNA binding shifts the population predominantly into one conformer such that the peaks from the depleted population are no longer observed. Analogous population shifts caused by partner binding or regulatory modifications have been observed for nucleic acid binding proteins (e.g. Ets-1) [59,60]. The second scenario is that nucleic acid binding alters local kinetics for a subset of residues, moving them into the intermediate exchange regime, leading to line broadening that renders the corresponding amide cross peaks non-observable. Exchange-driven broadening on nucleic acid binding has been reported in several systems, including homeodomain proteins interacting with nonspecific DNA [61], DNA-bound p53 [62], and viral systems where RNA conformational dynamics or protein–RNA remodeling are associated with μs–ms exchange signatures (e.g., HIV-1 RRE RNA dynamics and SARS-CoV-2 nucleocapsid NTD RNA binding) [63,64]. The observation that TCEP-induced mild reduction produces a 2D ^1^H–^15^N HSQC spectrum similar to that of the ssDNA-bound protease (Fig. 4B) requires additional study to clarify the mechanism involved.

Further, nsP2^pro^ was investigated to specify possible nucleic acid binding regions at the protein surface, using the PROBind webserver [41]. Predicted residues involved in DNA and RNA interaction differ in quantity and the localization in the protein. 14 amino acid residues were predicted to interact with DNA and 23 residues with RNA. DNA binding regions are exclusively localized at the MTase domain, also the majority of the RNA binding residues, but the protease domain carries five amino acids with the potential to interact with RNA. The proposed residues involved in DNA interaction included two main regions _746_RSS_749_ and _770_DNGRR_774_. Contrary, predicted regions for RNA interaction included _773_RRN_775_, _706_QML_708_, _648_TKR_650_, _630_K(I)NGH_634_ (predicted without the I) and five residues in the protease domain, _519_NE_520_, _515_E and _512_YS_513_ (Fig. S16).

Viral proteins utilize various structural nucleic acid binding motifs, including the RNA recognition motif (RRM) and the zinc finger motif (Frequently involved in DNA binding), to interact with viral and host nucleic acids. Moreover, arginine plays an important role in several RNA binding motifs in virus proteins, e.g. arginine rich motif (ARM) [65], HR motif [66], RG/RGG motif [67], SR/RS motif [68]. Arginine has a high occurrence in the predicted nucleic acid interaction regions of nsP2^pro^, nevertheless these regions differs in the sequence and structure to the typical motifs mentioned above. Viral proteins often exhibit significant variation in their RNA and DNA binding motifs. This variation arises from evolutionary pressures and the need for viruses to efficiently interact with the host cell's RNA machinery and evade the host's immune system [69]. However, arginine plays a crucial role in nucleic acid binding to its ability to form electrostatic interactions with the phosphate backbone and base pairs in nucleic acids [70]. Contrary, the similar activation observed with random ssDNA and RNA sequences, together with the sensitivity of this effect to increasing ionic strength, suggests that electrostatic interactions represent a key driving force for nucleic acid-mediated activation of nsP2^pro^. These observations indicate that electrostatic contacts are sufficient to promote activation in vitro, while sequence- or structure-specific interactions may provide additional layers of regulation under physiological conditions. Given the highly RNA-enriched environment of the viral replication complex, weak electrostatic interactions could facilitate the recruitment or positioning of nsP2^pro^, whereas specific viral RNA motifs or structural elements may further fine-tune its activity. Moreover, several studies demonstrate that non-specific electrostatic forces primarily drive viral protease and nucleic acid interactions, this was shown for the proteases of HIV-1, human adenovirus, foamy virus, picornavirus and seneca valley virus [23,27,30,71,72]

The MD simulations performed in this study demonstrated a stable interaction of RNA and DNA with the nsP2^pro^ Mtase domain for each replicate. These interactions induce a conformational movement in the active site of the protease from a closed to an open state. A previous study from our group demonstrated an interdomain motion leading to closed and open conformations of the nsP2^pro^ active site. Thereby, a flap formed by residues A545 to H548 can adopt positions either close to or far from the residues _667_NLELGL_672_ in the MTase domain. When the active site adopts a more open conformation, the flap moves farther away from the loop on the C-terminal domain [32]. Nearby the MTase _667_NLELGL_672_ loop are predicted nucleic binding regions are located. Especially, _706_QML_708_ is in direct neighborhood of this loop, Met707 and Leu708 have a distance of 3.4 to 3.5 Å to the _667_NLELGL_672_ loop, which makes the formation of van der waals (VDW) interactions between both regions highly possible (Figs. S16-S17). VDW interactions occur at distances typically between 2.5 and 4.6 angstroms (Å), averaging around 3.6 Å [73]. We assume that nucleic acids may interact with _706_QML_708_ and disturb the interaction to _667_NLELGL_672_, with the result that the active site exists in the open conformation. Moreover, the residues _519_NE_520_, _515_E and _512_YS_513_ are located at a helix beside the active site. Nucleic acid binding in this region may induce a conformational change that results in the open conformation of the protease active site, which may explain the increased activity of CHIKV nsP2^pro^ after nucleic acid binding.

Computational alanine scanning further revealed that individual arginine residues contribute unequally to nucleic acid recognition within the proposed binding interface of CHIKV nsP2^pro^, with their relative contributions ranking as R678 > R749 > R746 > R774 > R773. Moreover, several substitutions, including R678A and S747A, displayed differential effects on DNA and RNA binding, indicating that this region may contribute to nucleic acid discrimination. Whether these residues are involved in regulating nsP2^pro^ activity or selectively mediating DNA- versus RNA-dependent activation remains an intriguing question that warrants future biochemical and structural investigation.

Remarkably, substitutions N768A and F769A produced the largest reductions in predicted binding affinity, identifying these residues as potential interaction hotspots within the nucleic acid-binding interface. This observation is consistent with established roles of asparagine and phenylalanine residues in nucleic acid recognition across diverse protein families. Asparagine side chains frequently mediate sequence-specific recognition through stereospecific hydrogen-bonding interactions with exposed nucleobases [74,75], whereas phenylalanine residues stabilize protein–nucleic acid complexes through aromatic stacking interactions with nucleobases or contacts with the sugar-phosphate backbone that can promote local base unstacking and nucleic acid bending [[75], [76], [77]]. Collectively, these findings suggest that N768 and F769 constitute key structural determinants of nucleic acid recognition and may contribute to the molecular mechanism underlying nucleic acid-dependent regulation of CHIKV nsP2^pro^.

## 5 Conclusion and future perspectives

In this study, it was demonstrated that single-stranded nucleic acids-including both DNA aptamers and structured RNA fragments-can significantly enhance the catalytic activity of the Chikungunya virus nsP2^pro^. This enhancement appears to be influenced by several factors: the single-stranded nature of the nucleic acid, a minimum oligonucleotide length, the presence of stable secondary structures, and sensitivity to ionic strength. Molecular docking analyses predicted that these nucleic acids interact with the methyltransferase domain of nsP2^pro^; however, this binding site remains to be experimentally validated. Further studies are needed to confirm the precise binding interface and determine whether this interaction occurs under physiological conditions. Future investigations should focus on assessing the in vivo relevance of these interactions during CHIKV infection and replication, as well as on high-resolution structural studies to define the molecular details of the nucleic acid–nsP2^pro^ complex.

## CRediT authorship contribution statement

**Mohammadamin Mastalipour:** Writing – review & editing, Writing – original draft, Investigation, Formal analysis, Data curation, Conceptualization. **Danilo Silva Olivier:** Writing – review & editing, Methodology, Investigation, Formal analysis, Data curation. **Mônika Apareçida Coronado:** Writing – review & editing, Formal analysis. **Andrew J. Dingley:** Writing – review & editing, Formal analysis, Data curation. **Ruth Anasthasia Siahaan:** Writing – review & editing, Investigation. **Alissa Drees:** Writing – review & editing, Methodology, Investigation, Data curation. **Christian Ahlers:** Writing – review & editing, Software, Investigation, Data curation. **Markus Fischer:** Writing – review & editing, Supervision, Resources. **Dieter Willbold:** Writing – review & editing, Resources. **Raphael Josef Eberle:** Writing – review & editing, Supervision, Conceptualization.

## Declaration of competing interest

The authors declare that they have no known competing financial interests or personal relationships that could have appeared to influence the work reported in this paper.

## Data Availability

Data will be made available on request.

## Appendix Supplementary materials

Image, application 1
