# Supplementary material for: Single-stranded nucleic acid binding enhances the in vitro catalytic activity of Chikungunya virus nsP2 protease
Source: BBA Adv. 2026 Jul 9;10:100198. doi: 10.1016/j.bbadva.2026.100198 (PMC13383954; doi:10.1016/j.bbadva.2026.100198)
Supplement: Supplementary file 1 [file mmc1.docx]

**Single-stranded nucleic acid binding enhances the *in vitro* catalytic activity of Chikungunya virus nsP2 protease**

Mohammadamin Mastalipour ^a^, Danilo Silva Olivier ^b^, Mônika Apareçida Coronado ^a^, Andrew J. Dingley ^a,c^ , Ruth Anasthasia Siahaan ^d^, Alissa Drees ^e^, Christian Ahlers ^e^, Markus Fischer ^e^, Dieter Willbold ^a^, Raphael Josef Eberle ^f, *^

^a^ Institut für Physikalische Biologie, Heinrich-Heine-Universität Düsseldorf, Düsseldorf, Germany

^b^ Integrated Sciences Center, Campus Cimba, Federal University of Tocantins, Araguaína 77824-838, TO, Brazil.

^c^ Institut für Biologische Informationsprozesse (IBI-7), Forschungszentrum Jülich, Jülich, Germany

^d^ Faculty of Chemistry and Biotechnology, FH Aachen, Campus Jülich, Germany

^e^ Hamburg School of Food Science, Institute of Food Chemistry, University of Hamburg, Hamburg, Germany

^f^ Institut für Biochemische Pflanzenphysiologie, Heinrich-Heine-Universität Düsseldorf, Düsseldorf, Germany

* Correspondence to**:** [eberler@hhu.de](mailto:eberler@hhu.de)

**Table of content**

**Supplementary figure S1.** Primary data of CHIKV nsP2^pro^ activity assay.

**Supplementary figure S2.** Structural comparison of the nsP2^pro^ structure and the generated nsP2^pro^ AlphaFold 3 model.

**Supplementary figure S3.** K_D_ fitting of DNA aptamers DAC1–DAC10.

**Supplementary figure S4.** Predicted secondary Structures of DAC1–DAC5.

**Supplementary figure S5.** Predicted secondary Structures of DAC6–DAC10.

**Supplementary figure S6.** Secondary structure of DAC1–DAC4.

**Supplementary figure S7.** Secondary structure of DAC5–DAC8.

**Supplementary figure S8.** Secondary structure of DAC9–DAC10 and RAC1-RAC2.

**Supplementary figure S9.** Amino acids of predicted nucleic acid binding regions in the nsP2^pro^ sequence.

**Supplementary figure S10.** Coordination of RNA and DNA at the nsP2^pro^ Mtase domain in models generated by AlfaFold.

**Supplementary figure S11.** RMSD, RMSF, RoG and surface area profiles for duplicates of 200 ns of MD simulations of CHIKV nsP2^pro^-RNA complex.

**Supplementary figure S12.** RMSD, RMSF, RoG and surface area profiles for duplicates of 200 ns of MD simulations of CHIKV nsP2^pro^-DNA complex.

**Supplementary figure S13.** Numbers of H-bonds between the nucleic acids and CHIKV nsP2^pro^ residues during the 200 ns MD simulations.

**Supplementary figure S14.** CHIKV nsP2^pro^ active site movement after MD simulations with and without nucleic acids for two independent simulations.

**Supplementary figure S15.** **Control experiments confirm substrate-specific fluorescence signal in the nsP2^pro^ activity assay.**

**Supplementary figure S16.** **Surface view of nsP2^pro^ with labeled predicted nucleic acid binding areas, protease active site and MTase loop _1202_NLELG_1206_.**

**Supplementary figure S17.** **Closed conformation of the nsP2^pro^ active site with _1202_NLELG_1206_ and possible _1241_QML_1243_ interactions.**

**Supplementary Table S1**. Sequences and properties of the RNAs used in this study.

**Supplementary Table S2**. CHIKV nsP2^pro^ amino acid residues involved in the interaction with DNA and RNA.

**Supplementary Table S3.** Results of the *in silico* alanine scanning.


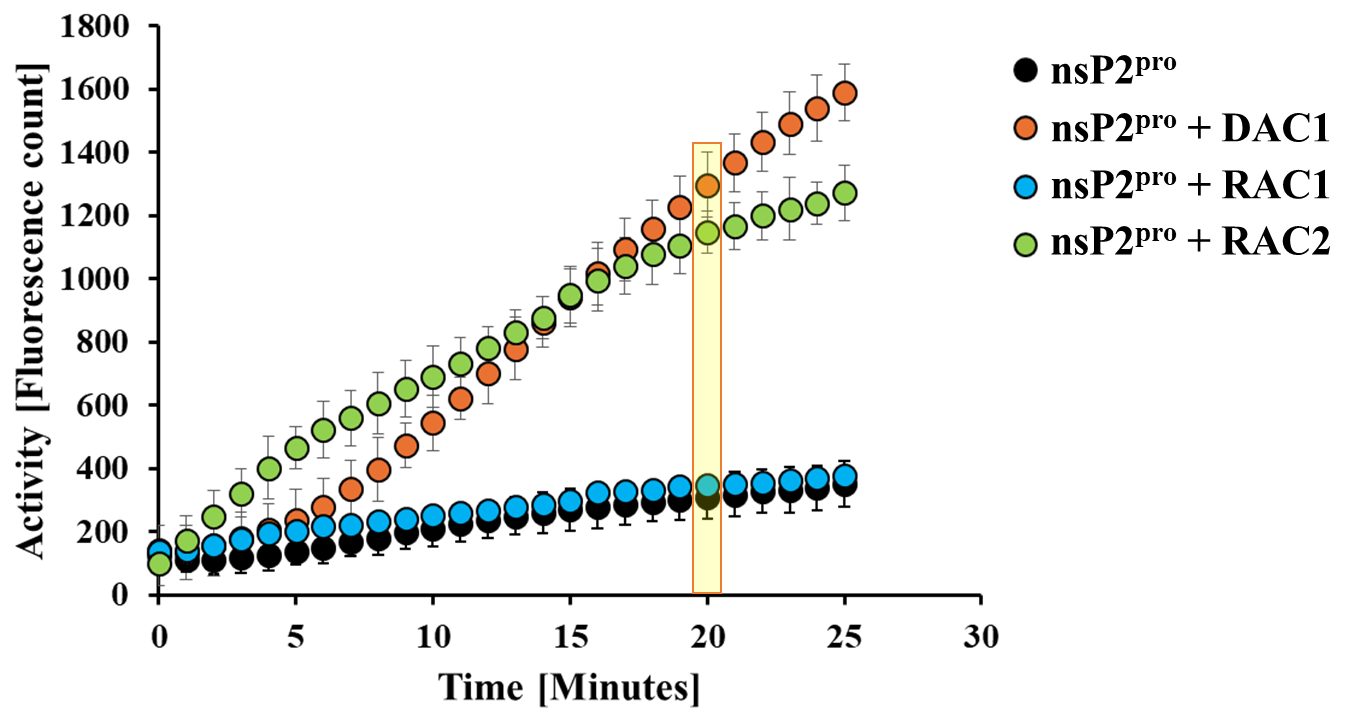


**Fig. S1. Primary data of CHIKV nsP2^pro^ activity assay.** Activity data for the protease alone as well as for the protease in the presence of either aptamer (DAC1) or RNA (RAC1 and RAC2). The Yellow box mark 20-minute time point for comparative analysis.

**
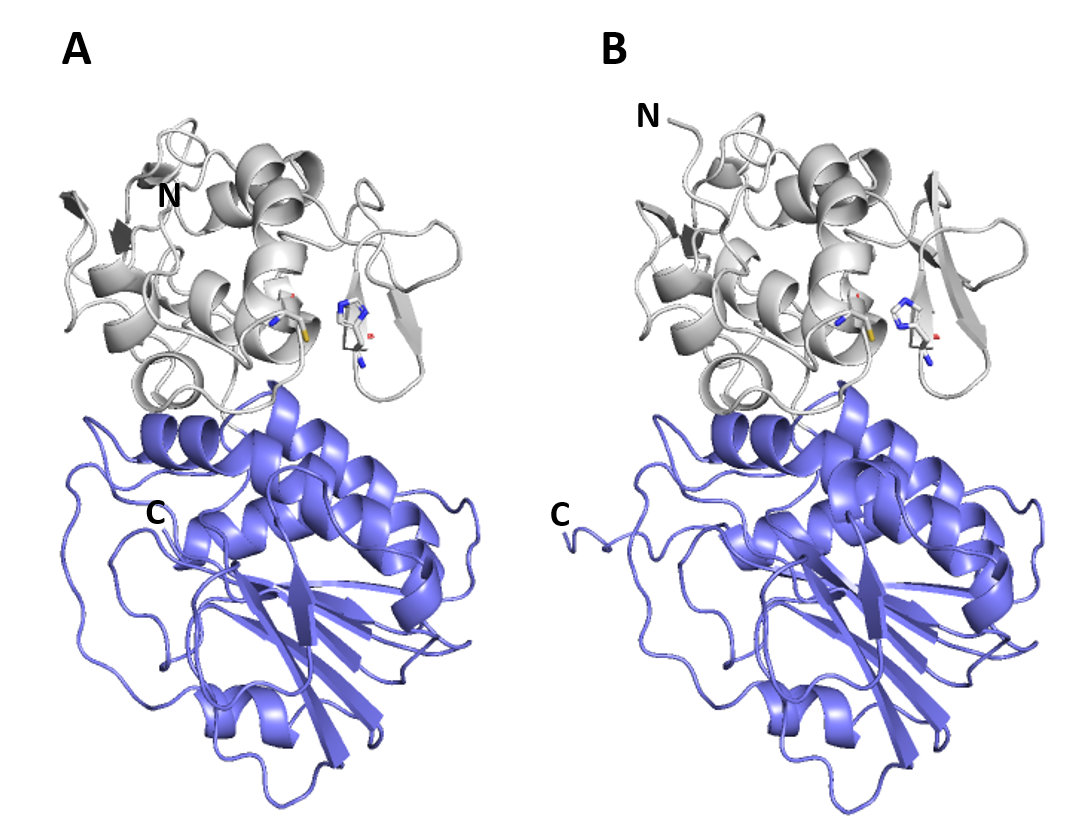
**

**Fig. S2.** **Structural comparison of the nsP2^pro^ structure and the generated nsP2^pro^ AlphaFold model.** Both structures are shown in ribbon view, the papain-like cysteine protease is colored in grey and the Ftsj methyltransferase (MTase)-like domain in blue. An overlay of both structures indicated a RMSD value of 0.435 (2165 to 2165 atoms). **(A)** nsP2^pro^ crystal structure (PDB code: 3TRK) and **(B)** nsP2^pro^ AlphaFold 3 model.

**
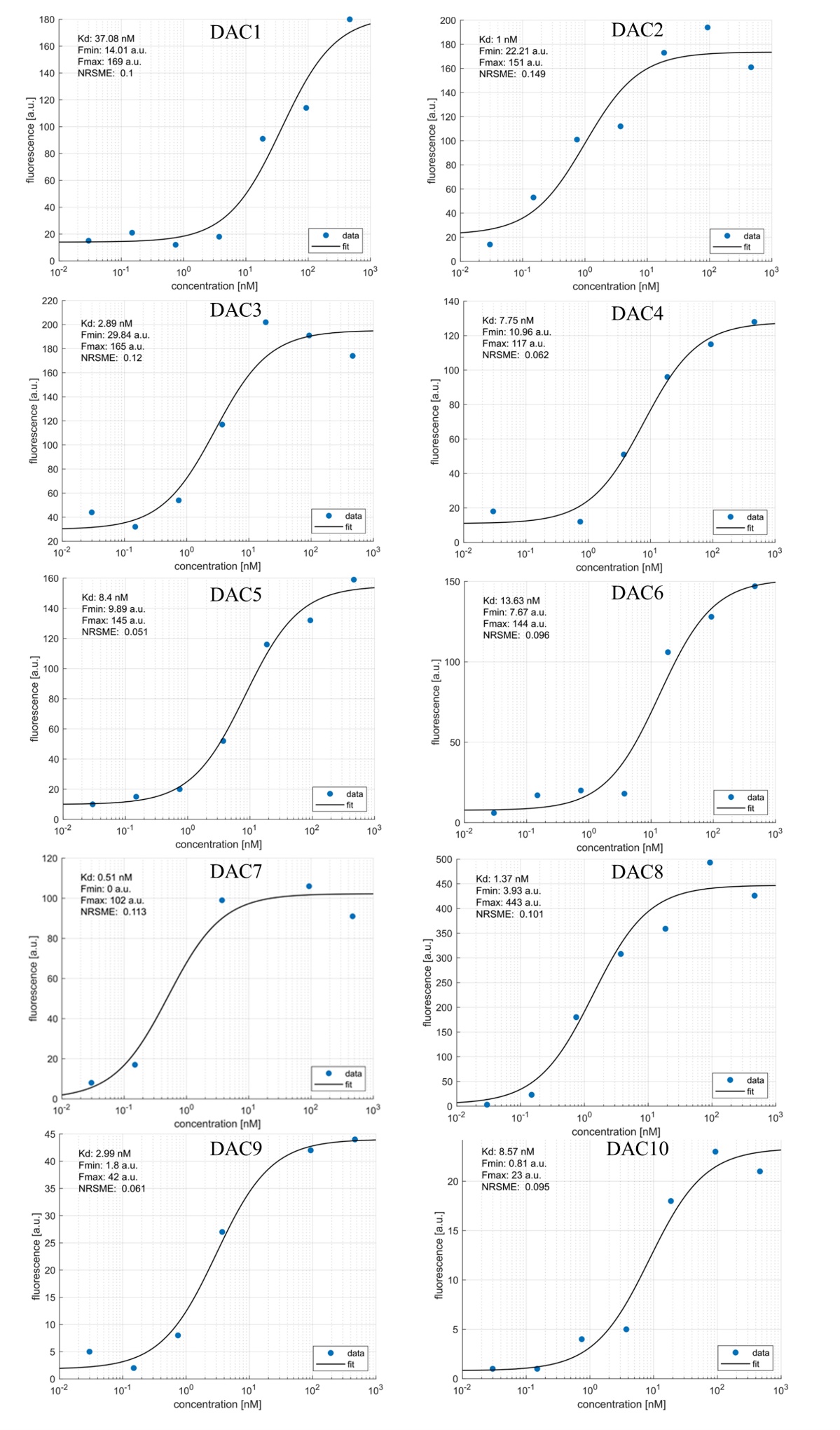
**

**Fig. S3.** **K_D_ fitting of DNA aptamers DAC1–DAC10**. Binding affinities for DAC1–DAC10 were determined using HiTS-FLIP, and the data were fitted using a nonlinear Hill-fit.

**
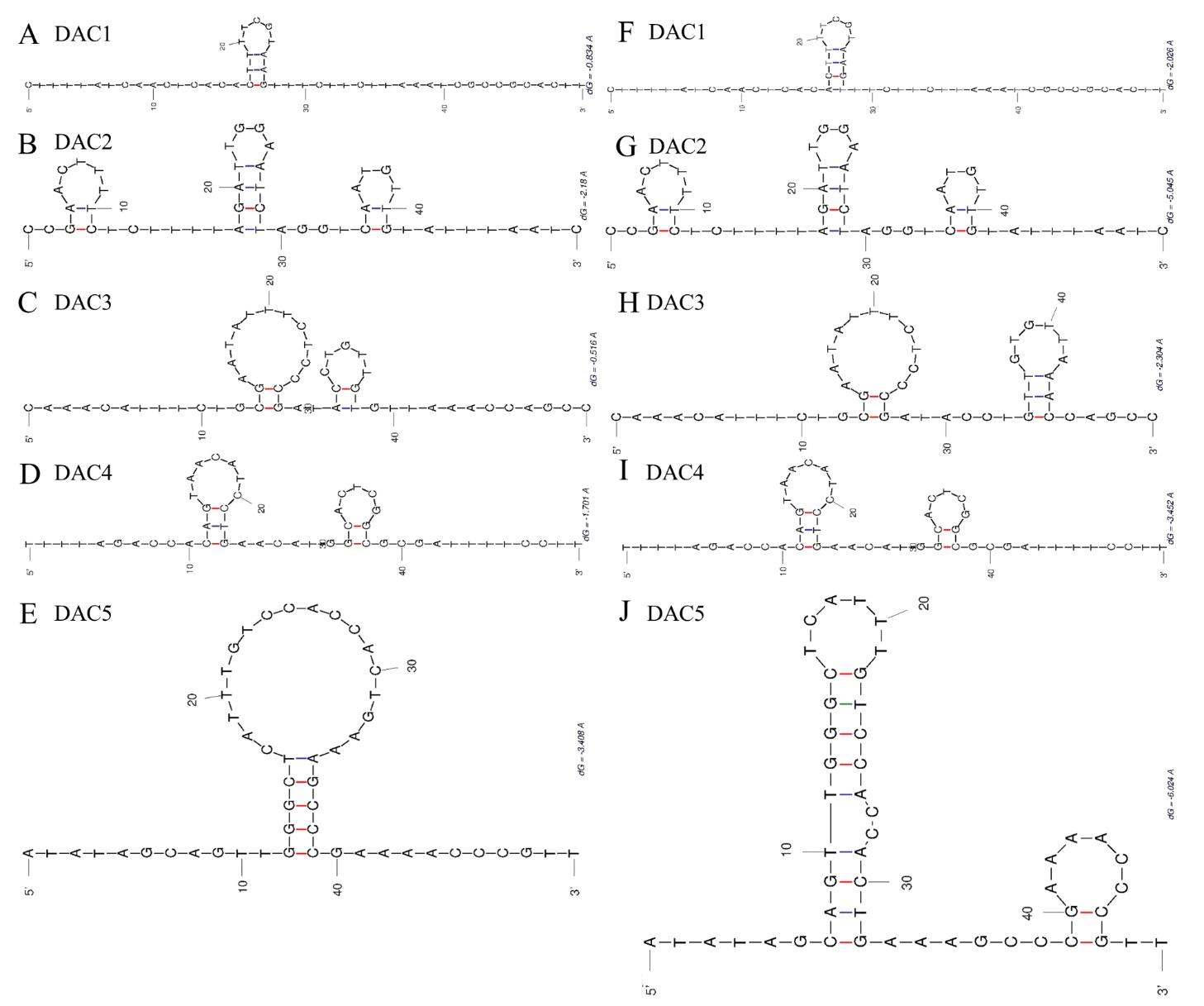
**

**Fig. S4. Predicted secondary structures of DAC1–DAC5.** Panels (A–E) show the predicted secondary structures of DAC1–DAC5 in water at 18°C, and panels (F–J) display the corresponding structures in 1×PBS at 18°C. Predictions were generated using the DINAMelt Server – Quikfold web tool, and only the structure with the lowest ΔG (Gibbs free energy) was presented.

**
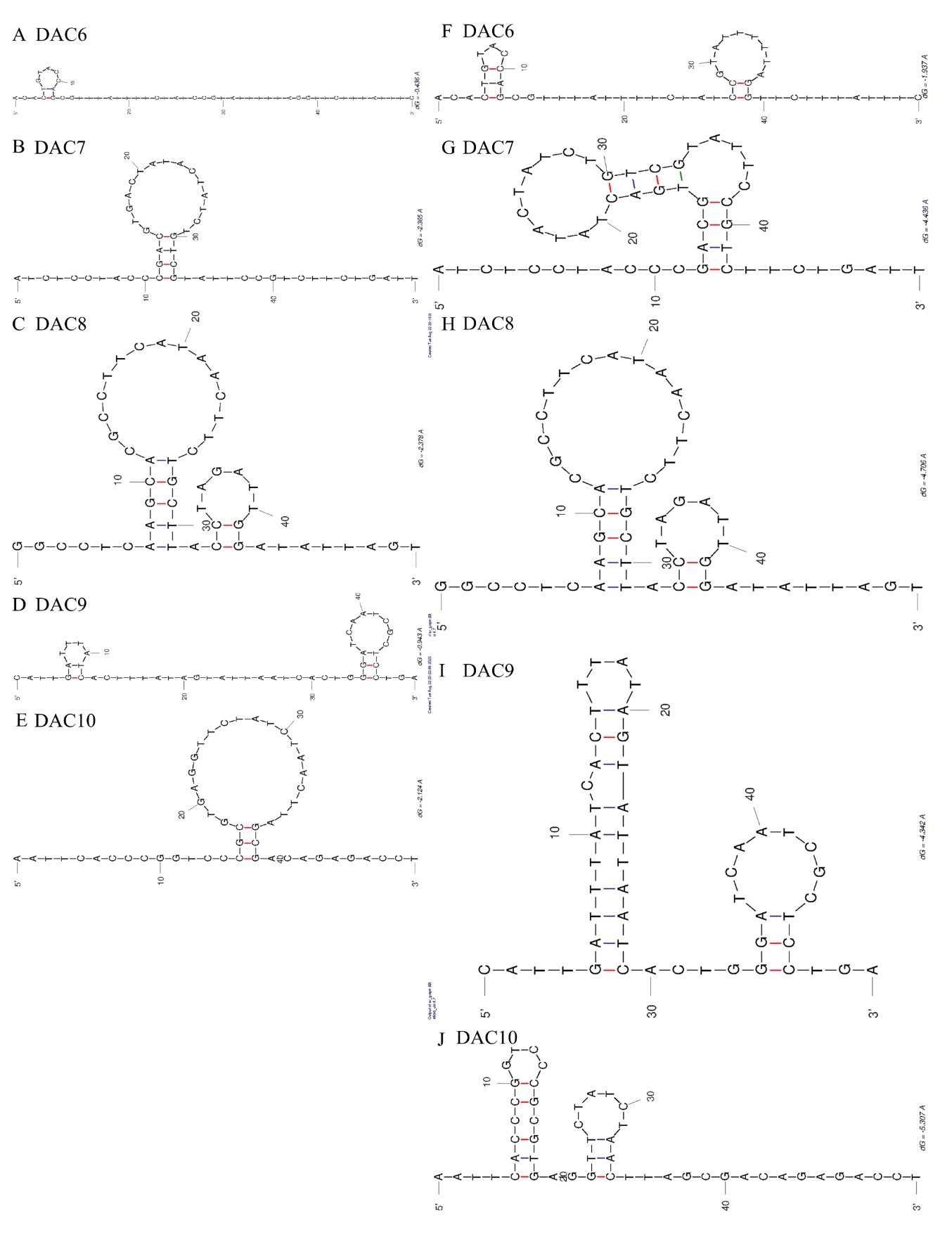
Fig. S5. Predicted secondary structures of DAC6–DAC10.** Panels (A–E) show the predicted secondary structures of DAC6–DAC10 in water at 18°C, and panels (F–J) display the corresponding structures in 1×PBS at 18°C. Predictions were generated using the DINAMelt Server – Quikfold web tool, and only the structure with the lowest ΔG (Gibbs free energy) was presented.

**
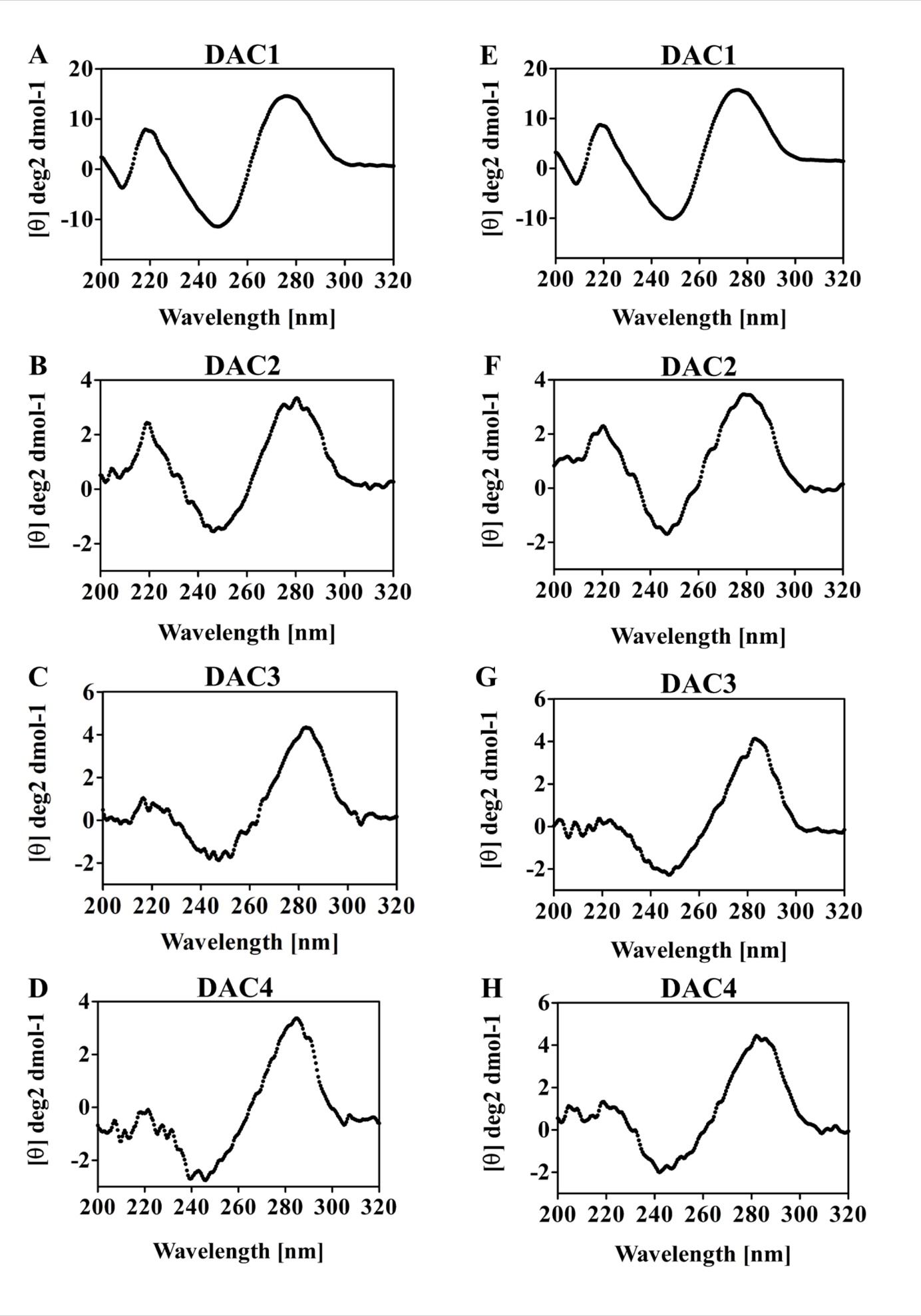
**

**Fig. S6**. **Secondary structure of DAC1–DAC4.** Panels (A–D) show the CD spectra of DAC1–DAC4 in water at 18°C, while panels (E–H) present the corresponding spectra in 1×PBS at 18°C. All spectra were recorded over the wavelength range of 320–200 nm

**
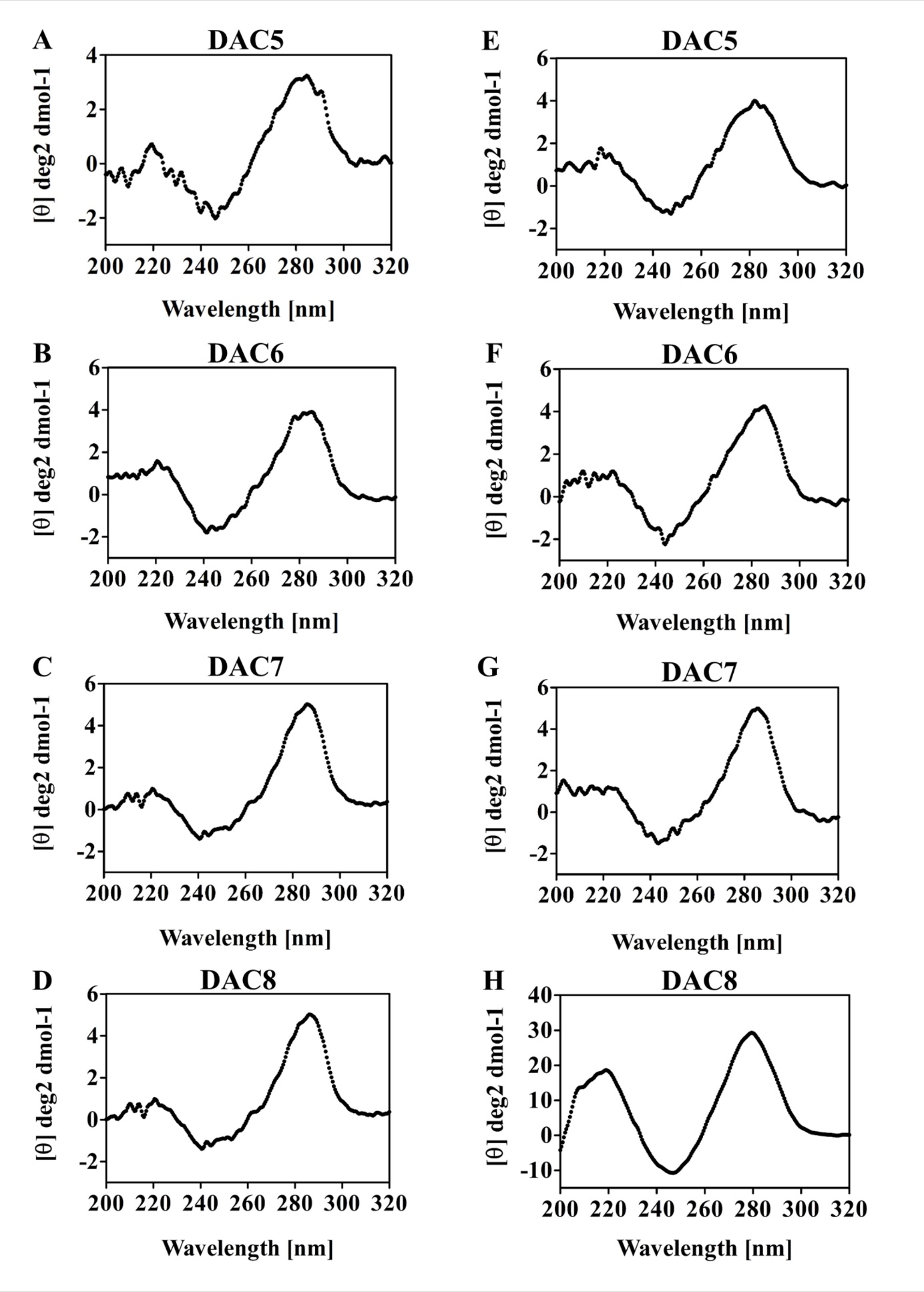
**

**Fig. S7**. **Secondary structure of DAC5–DAC8.** Panels (A–D) show the CD spectra of DAC5–DAC8 in water at 18°C, while panels (E–H) present the corresponding spectra in 1×PBS at 18°C. All spectra were recorded over the wavelength range of 320–200 nm

**
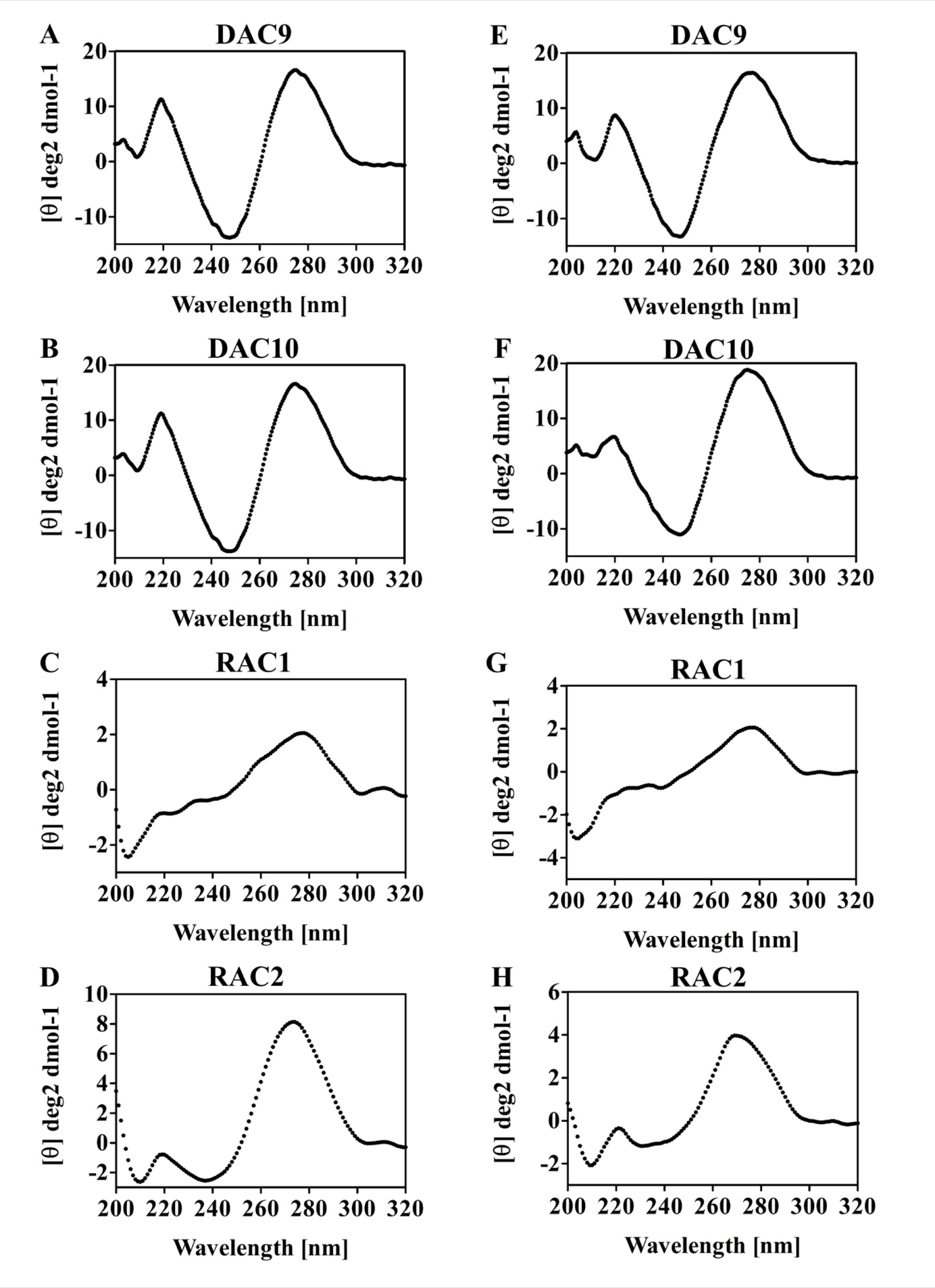
**

**Fig. S8**. **Secondary structure of DAC9–DAC10 and RAC1-RAC2.** Panels (A–D) show the CD spectra of **DAC9–DAC10 and RAC1-RAC2** in water at 18°C, while panels (E–H) present the corresponding spectra in 1×PBS at 18°C. All spectra were recorded over the wavelength range of 320–200 nm


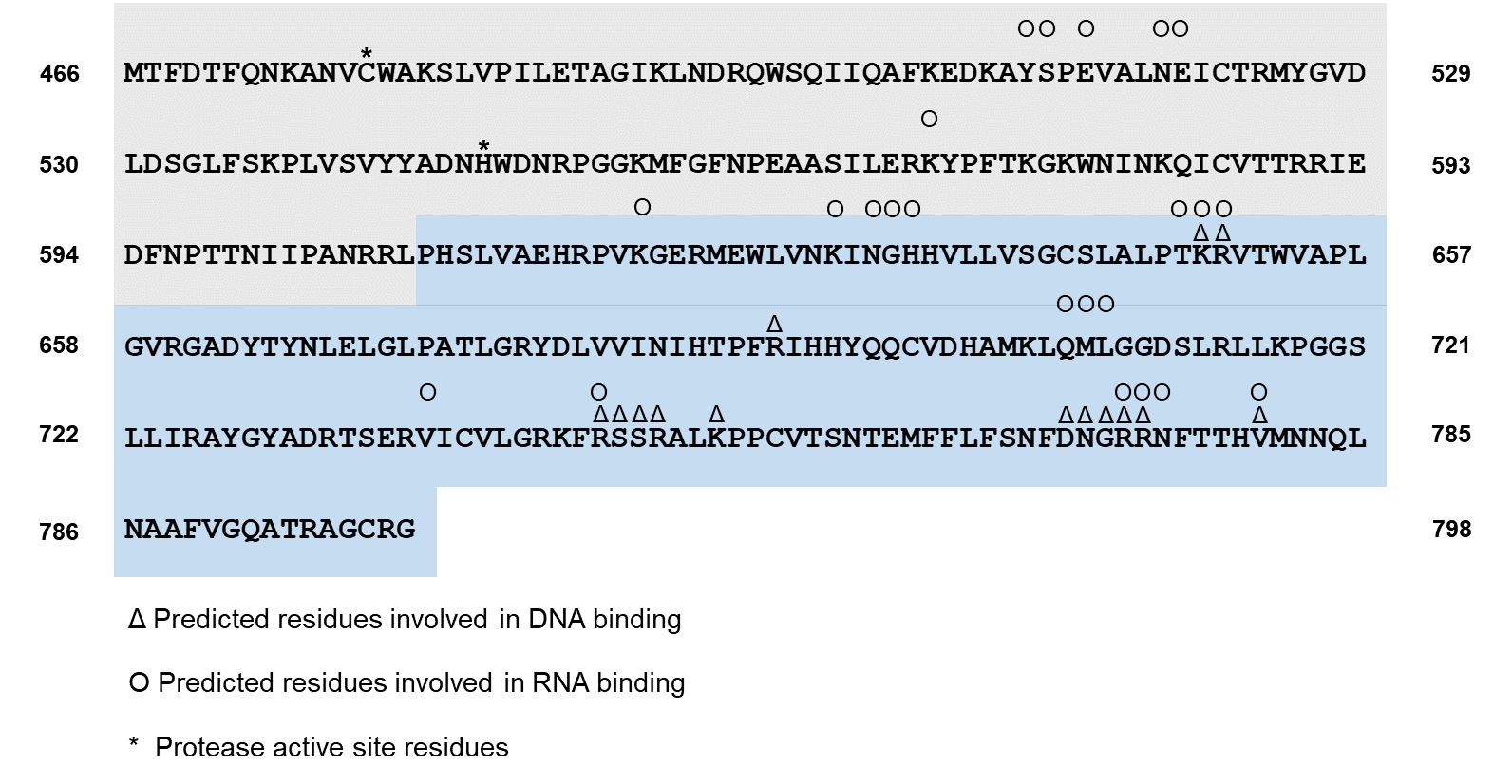


### Fig. S9. Predicted nucleic acid binding regions in the nsP2^pro^ sequence. The papain-like cysteine protease sequence is displayed with a grey background and the Ftsj methyltransferase (MTase)-like domain is highlighted in blue. The sequence numbering based on the CHIKV polyprotein. Predicted amino acids involved in DNA binding are labeled by a triangle and those predicted to be involved in RNA binding are labeled by a circle. The catalytically active residues of the cysteine protease are labeled by asterisks.

###
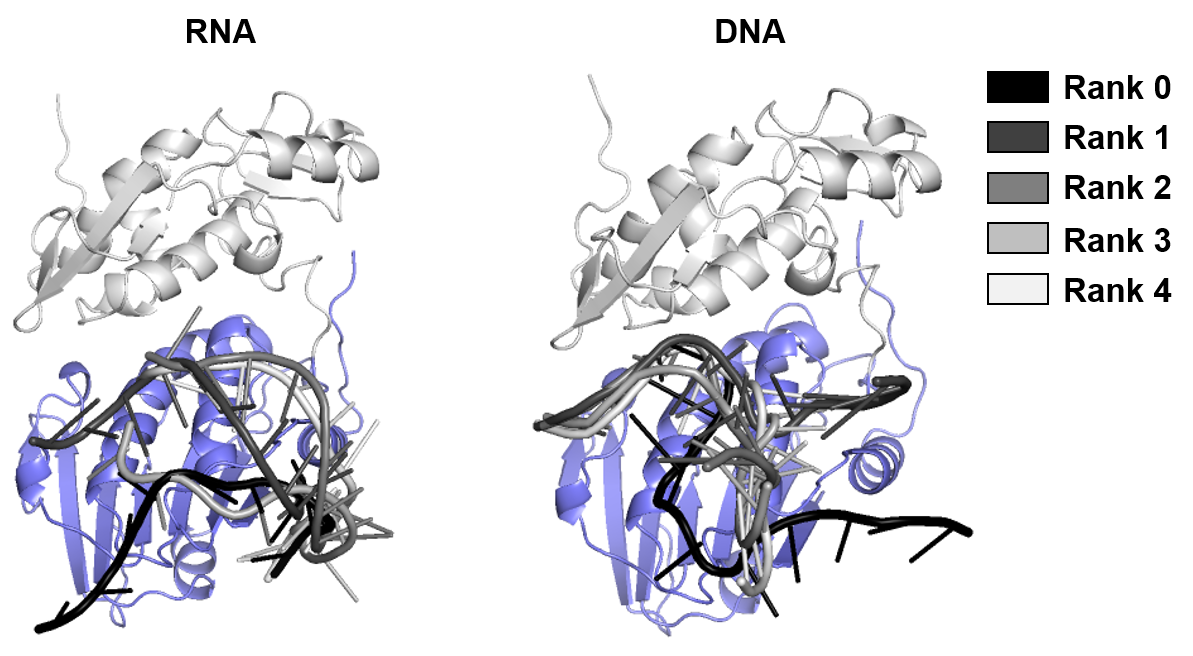


### Fig. S10. Coordination of RNA and DNA at the nsP2^pro^ Mtase domain in models generated by AlfaFold. The protease domain is colored in grey and the Mtase domain in blue. For RNA and DNA an overlay of the five models generated by AlfaFold are shown.

###
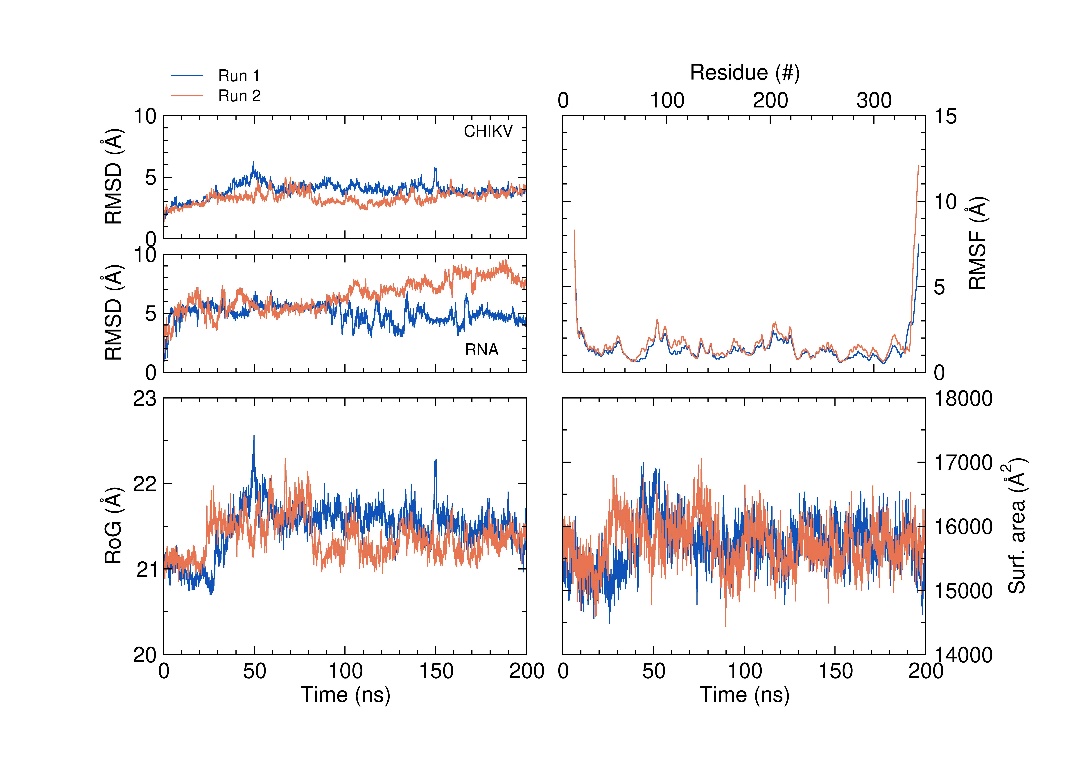


### Fig. S11. RMSD, RMSF, RoG and surface area profiles for duplicates of 200 ns of MD simulations of CHIKV nsP2^pro^-RNA complex. The RMSD time profiles concerning all backbone atoms of nsP2^pro^ and the RNA. The RMSF plot corresponds to the protease/RNA complex, the replicates are specified.

###
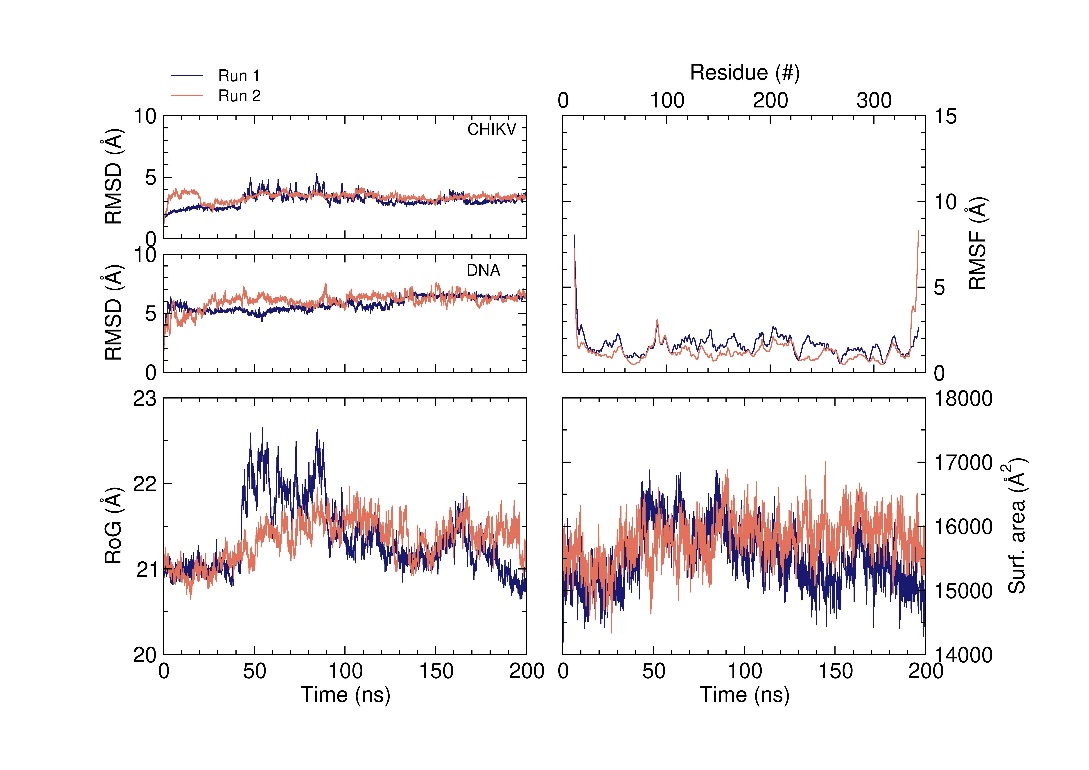


### Fig. S12. RMSD, RMSF, RoG and surface area profiles for duplicates of 200 ns of MD simulations of CHIKV nsP2^pro^-DNA complex. The RMSD time profiles concerning all backbone atoms of nsP2^pro^ and the DNA. The RMSF plot corresponds to the protease/DNA complex, the replicates are specified.

###
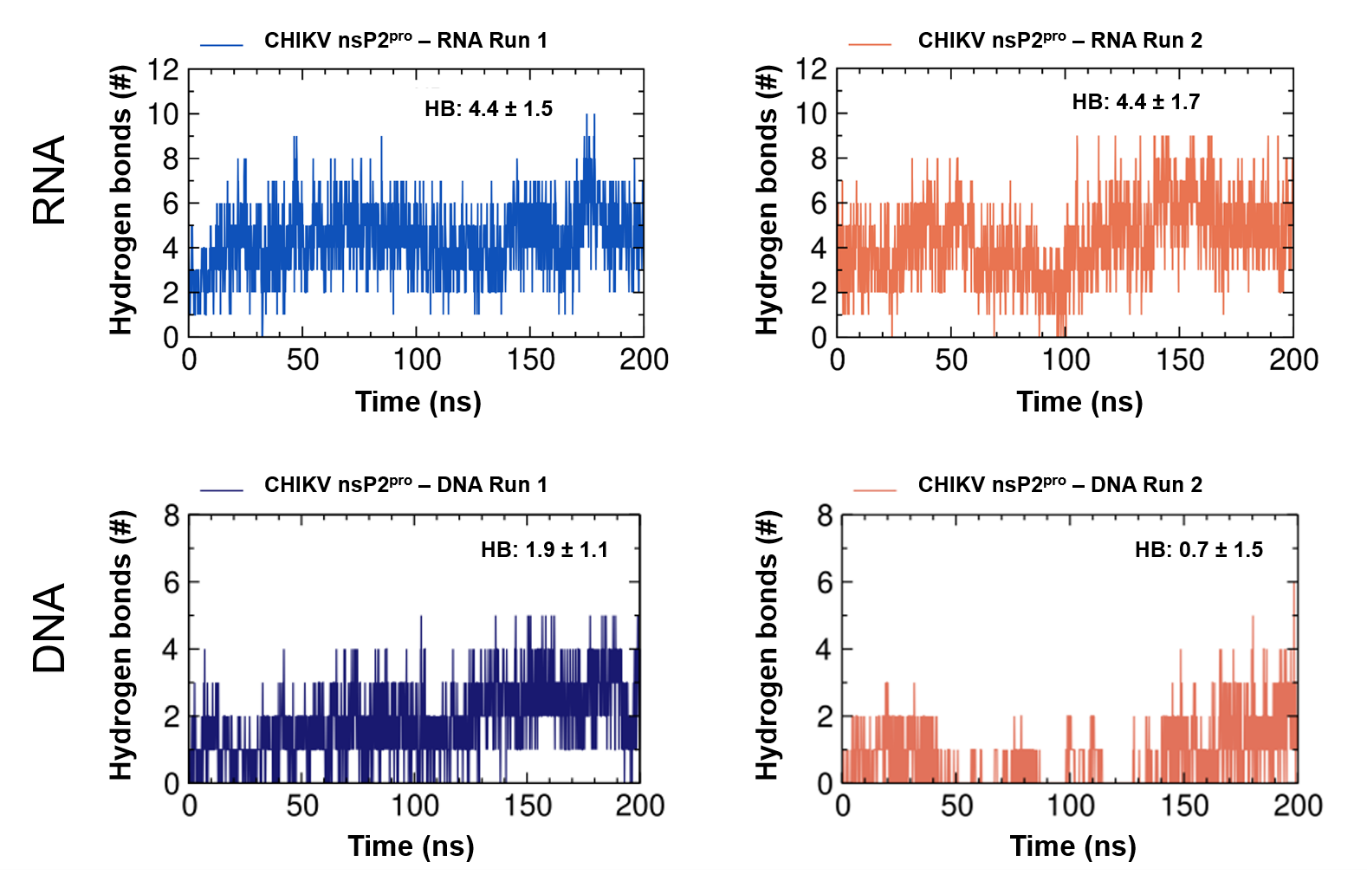


### Fig. S13. Numbers of H-bonds between the nucleic acids and CHIKV nsP2^pro^ residues during the 200 ns MD simulations. For each replicate the H-bonds are shown. The general criteria to determine the occurrence of the H-bond was a donor receptor distance ≤ 3.5 Å and a donor-H-acceptor angle ≥ 120°. The analyses represents the average number of intermolecular H-bonds formed algong each trajectory.

###
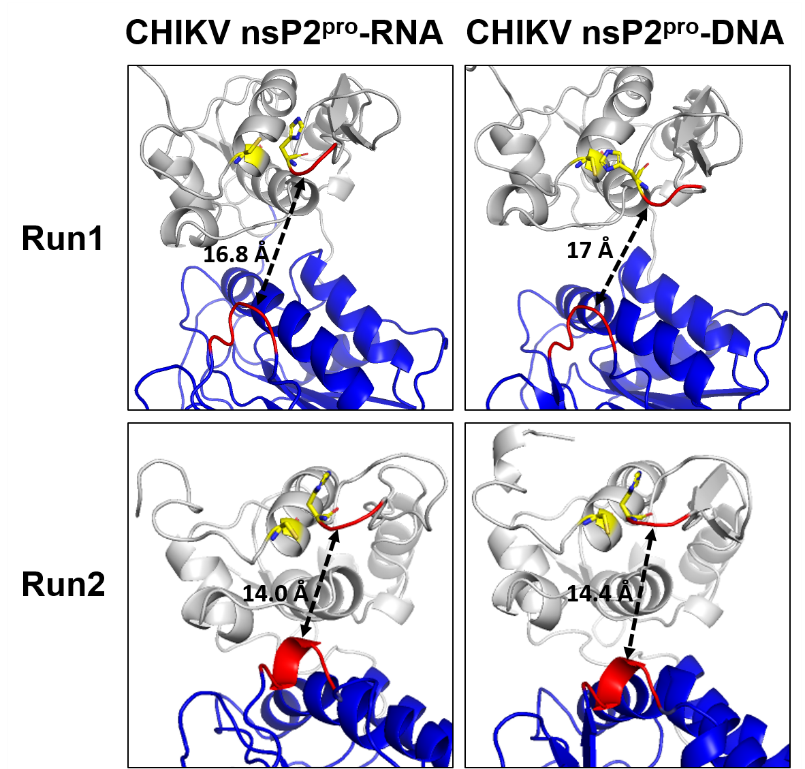


### Fig. S14. CHIKV nsP2^pro^ active site movement after MD simulations with and without nucleic acids for two independent simulations. The active site region is shown in ribbon view. The protease domain is colored in grey, the Mtase domain in blue, the catalytical residues (Cys478 and His548) in yellow, the protease active site loop and the Mtase loop _667_NLELGL_672_ are colored in red and the volume in orange. The distance in Å is shown between the protease active site loop carrying His548 and the Mtase loop.


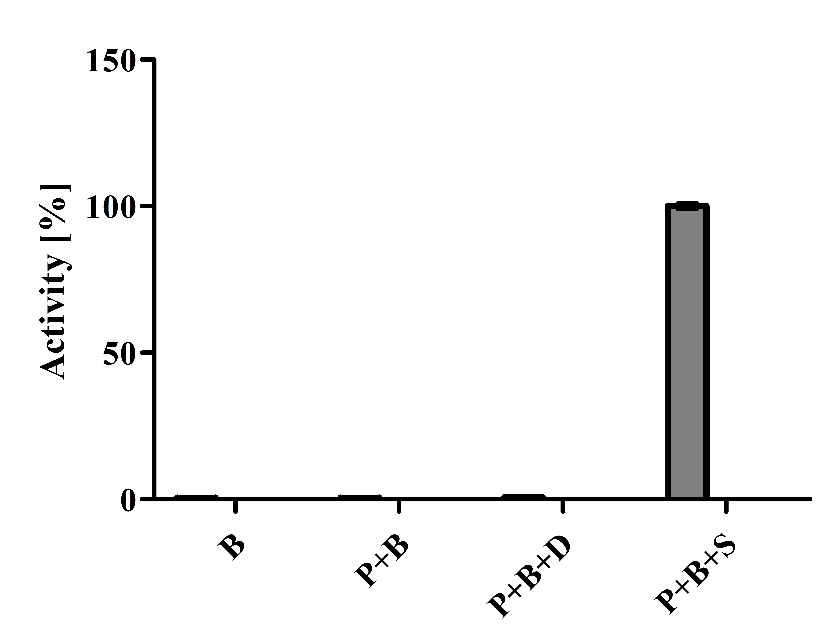


### Fig. S15. ****Control experiments confirm substrate-specific fluorescence signal in the nsP2^pro^ activity assay**.** Fluorescence intensity was measured under four conditions to evaluate potential background signal in the absence of the fluorogenic substrate: (1) buffer alone (B), (2) buffer with CHIKV nsP2^pro^ (P), and (3) buffer with both protease and DNA aptamer DAC8 (D). A positive control containing buffer, protease, and the fluorogenic substrate (Control) was included to represent true enzymatic activity. No significant fluorescence was detected in the absence of the substrate, confirming that background signal from buffer components, protease, or DNA aptamer was negligible.

**
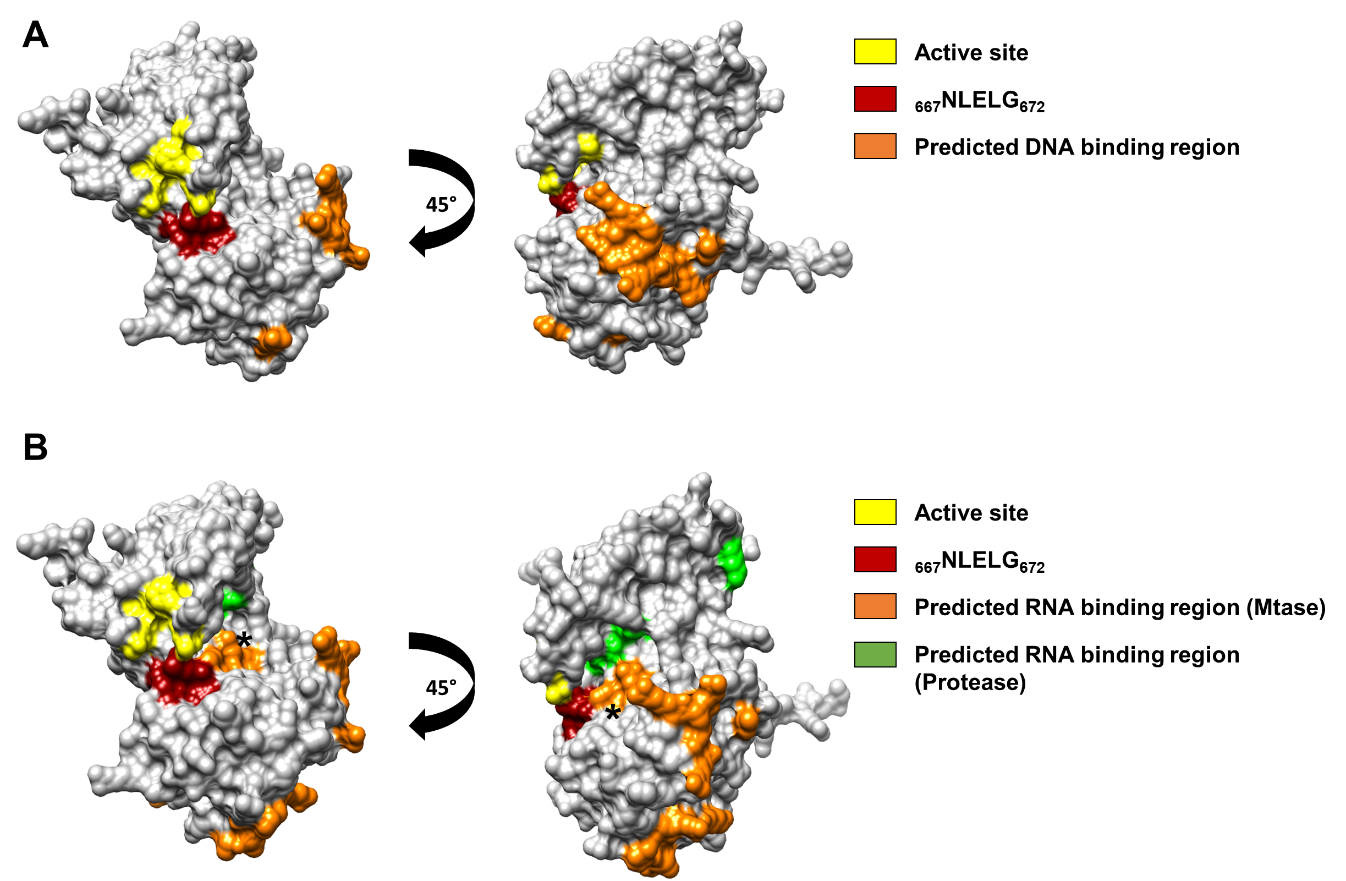
**

### Fig. S16. ****Surface view of nsP2^pro^ with labeled predicted nucleic acid binding areas, protease active site and MTase loop _667_NLELG_672_.** The nsP2^pro^ active site is shown in close conformation were the protease active site residues Cys478 and His548 and the Mtase loop _667_NLELG_672_ are nearby. A: Predicted DNA binding region. B: Predicted RNA binding region. Asterisk label the position of _706_QML_708_.**

**
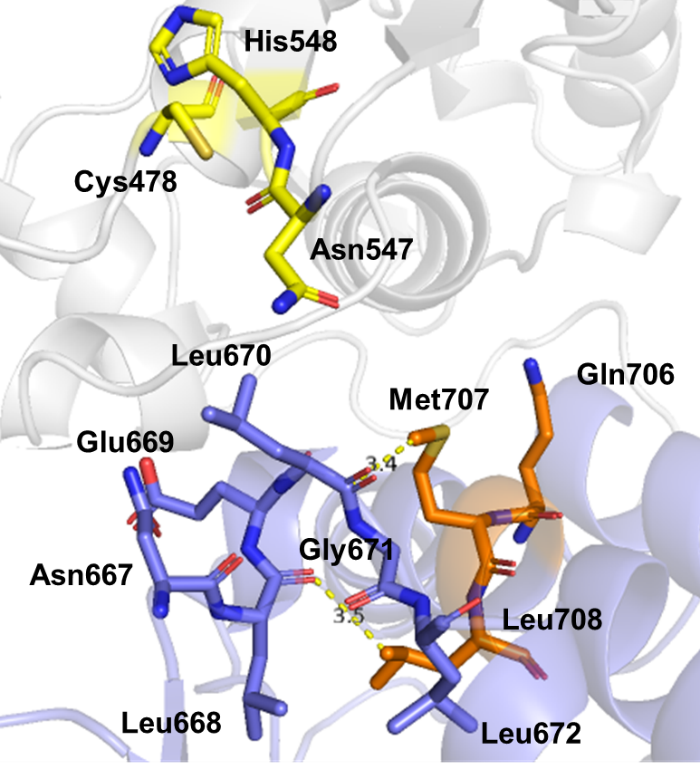
**

### Fig. S17. ****Closed conformation of the nsP2^pro^ active site with _667_NLELGL_672_ and possible _706_QML_708_ interactions.** The nsP2^pro^ active site residues are colored in yellow. The Mtase loop _667_NLELGL_672_ is colored in blue and the Mtase region _706_QML_708_ is colored in orange.**

**Table S1.** Sequences and properties of the RNAs used in this study. The RNA sequences were derived from the Chikungunya virus (CHIKV) genome (associated GenBank accession: **KM673291**).

| RNA | Sequence (5'-3') | Origin | Tm [°C] | Manufacturer |
| --- | --- | --- | --- | --- |
| RAC1 | CGTUCG | CHIKV genome | 0.0 | Integrated DNA Technologies (IDT) |
| RAC2 | CGUCGCUAUA | CHIKV genome | 21.1 | Integrated DNA Technologies (IDT) |

**Table S2.** CHIKV nsP2^pro^ amino acid residues involved in the interaction with DNA and RNA. Results of prediction using ProBind and after MD simulations (Residues interact with are shown RNA in red and DNA in yellow).

| **Residues in**  **nsP2^pro^** | **Prediction ProBind** | | **MD simulation RNA** | | **MD simulation DNA** | |
| --- | --- | --- | --- | --- | --- | --- |
|  | **RNA** | **DNA** | **Run1** | **Run2** | **Run1** | **Run2** |
| **Lys620** |  |  |  |  |  |  |
| **Lys630** |  |  |  |  |  |  |
| **Asn632** |  |  |  |  |  |  |
| **Gly633** |  |  |  |  |  |  |
| **His634** |  |  |  |  |  |  |
| **Thr648** |  |  |  |  |  |  |
| **Lys649** |  |  |  |  |  |  |
| **Arg650** |  |  |  |  |  |  |
| **Arg678** |  |  |  |  |  |  |
| **Arg691** |  |  |  |  |  |  |
| **Gln706** |  |  |  |  |  |  |
| **Met707** |  |  |  |  |  |  |
| **Leu708** |  |  |  |  |  |  |
| **Leu713** |  |  |  |  |  |  |
| **Arg714** |  |  |  |  |  |  |
| **Pro718** |  |  |  |  |  |  |
| **Arg736** |  |  |  |  |  |  |
| **Val737** |  |  |  |  |  |  |
| **Arg743** |  |  |  |  |  |  |
| **Arg746** |  |  |  |  |  |  |
| **Ser747** |  |  |  |  |  |  |
| **Ser748** |  |  |  |  |  |  |
| **Arg749** |  |  |  |  |  |  |
| **Lys752** |  |  |  |  |  |  |
| **Leu765** |  |  |  |  |  |  |
| **Asn768** |  |  |  |  |  |  |
| **Phe769** |  |  |  |  |  |  |
| **Asp770** |  |  |  |  |  |  |
| **Asn771** |  |  |  |  |  |  |
| **Gly772** |  |  |  |  |  |  |
| **Arg773** |  |  |  |  |  |  |
| **Arg774** |  |  |  |  |  |  |
| **Asn775** |  |  |  |  |  |  |
| **Phe776** |  |  |  |  |  |  |
| **Thr777** |  |  |  |  |  |  |
| **Val780** |  |  |  |  |  |  |
|  |  |  |  |  |  |  |
| **Amount of**  **residues** | **17** | **13** | **12** | **10** | **11** | **7** |

**Table S3.** Results of the *in silico* alanine scanning.

| **Residue/Model** | **DNA** | | **RNA** | |
| --- | --- | --- | --- | --- |
|  | **Run1** | **Run2** | **Run1** | **Run2** |
| **Wild Type** | -78.2 ± 10.2 | -57.8 ± 11.0 | -78.6 ± 12.4 | -78.3 ± 13.4 |
| **R678A** | -1.70 ± 2.00 | -0.39 ± 0.13 | -9.19 ± 3.97 | -3.16 ± 2.59 |
| **R746A** | -10.25 ± 4.05 | -9.53 ± 4.34 | -8.30 ± 5.30 | -8.26 ± 3.68 |
| **S747A** | 0.55 ± 1.06 | -0.93 ± 2.04 | -3.65 ± 3.04 | -4.95 ± 3.57 |
| **R749A** | -4.84 ± 1.99 | -0.96 ± 2.14 | -5.65 ± 3.16 | -5.45 ± 3.09 |
| **N768A** | 0.12 ± 0.22 | -1.16 ± 2.65 | 0.05 ± 0.22 | -4.28 ± 2.33 |
| **F769A** | -0.16 ± 0.44 | 0.04 ± 0.18 | -1.39 ± 0.65 | -0.42 ± 0.61 |
| **D770A** | -0.82 ± 4.54 | 0.38 ± 2.58 | -4.54 ± 5.87 | 2.62 ± 1.99 |
| **N771A** | -5.05 ± 2.65 | -1.19 ± 2.043 | -5.51 ± 1.61 | -2.41 ± 2.25 |
| **R773A** | -10.97 ± 3.08 | -10.58 ± 2.76 | -13.83 ± 1.68 | -7.35 ± 2.8 |
| **R774A** | -13.38 ± 2.73 | -4.91 ± 3.44 | -12.08 ± 4.71 | -10.12 ± 1.98 |
